# The expression and crystallization of Cry65Aa require two C-termini, revealing a novel evolutionary strategy of *Bacillus thuringiensis* Cry proteins

**DOI:** 10.1038/srep08291

**Published:** 2015-02-06

**Authors:** Dong-hai Peng, Cui-yun Pang, Han Wu, Qiong Huang, Jin-shui Zheng, Ming Sun

**Affiliations:** 1 State Key Laboratory of Agricultural Microbiology, College of life science and technology, Huazhong Agricultural University, Wuhan 430070, Hubei, People's Republic of China

## Abstract

The insecticidal crystal protein (Cry) genes of *Bacillus thuringiensis* are a key gene resource for generating transgenic crops with pest resistance. However, many *cry* genes cannot be expressed or form crystals in mother cells. Here, we report a novel Cry protein gene, *cry65Aa1*, which exists in an operon that contains a downstream gene encoding a hypothetical protein ORF2. We demonstrated that ORF2 is required for Cry65Aa1 expression and crystallization by function as a C-terminal crystallization domain. The *orf2* sequence is also required for Cry65Aa expression, because *orf2* transcripts have a stabilizing effect on *cry65Aa1* transcripts. Furthermore, we found that the crystallization of Cry65Aa1 required the Cry65Aa1 C-terminus in addition to ORF2 or a typical Cry protein C-terminal region. Finally, we showed that Cry65Aa1 has a selective cytotoxic effect on MDA-MB231 cancer cells. This report is the first description of a 130-kDa mass range Cry protein requiring two C-termini for crystallization. Our findings reveal a novel evolutionary strategy of Cry proteins and provide an explanation for the existence of Cry protein genes that cannot form crystals in *B. thuringiensis*. This study also provides a potential framework for isolating novel *cry* genes from “no crystal” *B. thuringiensis* strains.

*Bacillus thuringiensis* is a Gram-positive, spore-forming soil bacterium that can produce parasporal crystals during the sporulation phase. These parasporal crystals consist of proteins (Cry) that exhibit specific toxicity against a variety of insects, such as Lepidoptera, Coleoptera, and Diptera, and against some nematodes, mites, and protozoa^1^,^2^. Because of its strong and specific toxicity toward a wide range of insects, *B. thuringiensis* has been developed as a biopesticide and is the leading biopesticide for using as an alternative or supplement to synthetic chemical pesticides^3^. Additionally, the *cry* genes of *B. thuringiensis* are also considered to be a key gene resource for generating transgenic crops with pest resistance^4^.

However, because of the extensive and continuous usage of *B. thuringiensis*-based pesticides, some insect populations have developed resistance to *B. thuringiensis*^5^,^6^,^7^. Several strategies, such as the use of multiple toxins^8^, spatial or temporal refuge^9^, and high or ultrahigh doses^10^, are employed to delay the development of insect resistance to *B. thuringiensis*-based products. A major principle of management of resistance to insecticidal proteins is the use of combinations of different Cry proteins, especially proteins that have different receptors or different modes of action^11^. Therefore, the discovery of novel holotype crystal proteins in *B. thuringiensis* is considered to be one of the major approaches to overcoming potential insect resistance problems^1^,^11^. A series of techniques have been utilized to isolate novel *cry* genes, such as PCR hybridization^12^, PCR-restriction fragment length polymorphism (RFLP)^13^, and PCR product high-resolution melting (HRM) analysis^14^. The construction of *B. thuringiensis* DNA libraries in *Escherichia coli* and then screening by western blotting^15^ or hybridization^16^ has also been widely used for new *cry* gene isolation. Additionally, the development of DNA libraries in a crystalliferous mutant of *B. thuringiensis*, followed by microscopy observations, has been used to detect novel Cry protein genes^17^. Recently, next-generation sequencing technology has also been employed for the discovery of new *cry* genes^18^.

Using the above approaches, increasing numbers of crystal protein genes have been identified. Thus far, more than 700 *cry* genes from 73 classes have been cloned (http://www.lifesci.sussex.ac.uk/Home/Neil_Crickmore/Bt/). However, of the reported *cry* genes, some cannot be expressed or form crystals in *B. thuringiensis*^13^,^19^,^20^,^21^. As next-generation sequencing technology has been increasingly employed for the discovery of new *cry* genes, this kind of *cry* genes have been found more frequently^17^,^21^. Previously, we constructed a high-throughput system for isolating new *cry* genes by combining next-generation sequencing and a computational pipeline called BtToxin_scanner^21^. Using this method, we identified a large number of new *cry* genes. However, only a portion of these *cry* genes can be expressed in *B. thuringiensis*.

Why these *cry* genes cannot be expressed and form crystals in *B. thuringiensis* are not clear. Here, we report a novel 130-kDa mass range crystal protein gene, *cry65Aa1*, which was not expressed when introduced alone. We then found that *cry65Aa1* exists in an operon that encodes a hypothetical protein, ORF2, followed the Cry65Aa1 ORF. By contrast, *cry65Aa1* was expressed when co-expressed with *orf2*. We further demonstrated that ORF2 is required for Cry65Aa1 expression and crystallization and functions as an mRNA-stabilizing effector and a C-terminal crystallization domain. In addition, N-terminal or C-terminal replacement experiments demonstrated that the expression and crystallization of Cry65Aa1 require the Cry65Aa1 C-terminus and ORF2.

## Results

### Identification and cloning of a novel holotype crystal protein gene, *cry65Aa1*

**Figure 1:**
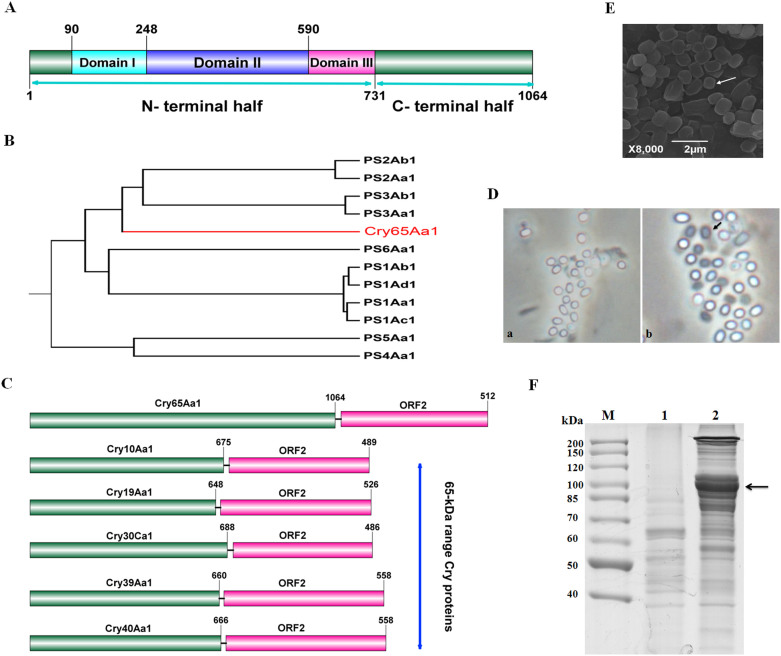
Cry65Aa1 is a novel holotype parasporin. (A), The phylogenetic dendrogram of parasporin proteins. (B), The schematic diagram of the secondary structure and domains of Cry65Aa1. The numbers on the top indicate the position of Cry65Aa1 amino acids. (C), Phase-contrast micrograph of crystals and spores. a, BMB171/pBMB1A-65A; b, BMB171/pBMB1331. (D). Total proteins were analyzed in 10% SDS- PAGE. M, protein molecular mass marker; Lane 1 and 2 showed the total proteins produced by BMB171/pBMB1A-65A, and BMB171/pBMB1331, respectively. (E), Scanning electron microscopy micrograph of Cry65Aa1 crystals produced by BMB171/pBMB1331. Magnification, ×8, 000. The position of Cry65Aa1 and crystals is indicated by arrows.

*B. thuringiensis* (strain SBT-003) has unique plasmid pattern and crystal morphology, so we finished its genome sequence to find novel *cry* genes (Genome accession No: AMYJ00000000). A novel *cry* sequence was identified in the SBT-003 genome sequence by using BtToxin_scanner^21^. This gene encodes a polypeptide of 1064 amino acid residues with a predicted molecular weight of 118,156.4 Da (Fig. S1) and has a typical three-domain-type structure with N- and C-terminal halves reminiscent of the 130-kDa mass range Cry proteins. Domain I has high similarity to the Endotoxin_N domain [pfam03945], and domain III has high similarity to the Delta_endotoxin_C domain [cd04085] (Fig. S2). The C-terminal domain of Cry65Aa1 consists of only 333 amino acids (with a molecular weight of 36.7 kDa), which is shorter than a typical C-terminal domain (approximately 55–65 kDa) (Fig. 1A). Sequence analysis indicates that the Cry65Aa1 C-terminal domain has low homology with a hypothetical protein (GenBank ID: EKU79138) from *Veillonella ratti* (Fig. S3).

Sequence alignment of the Cry65Aa1 protein with known Cry proteins revealed similarities within the 0–45% range. Therefore, the *B. thuringiensis* Nomenclature Committee designated the protein as a novel holotype Cry protein, Cry65Aa1. Cry65Aa1 exhibited the greatest similarity (28%) to Cry41Aa1 (accession No: AB116649) (Fig. S4), a parasporin protein (PS3Aa1) that exhibits cytocidal activity toward some cancer cells^22^. In the parasporin dendrogram, Cry65Aa1 proteins appeared grouped in a separate branch (Fig. 1B); thus, we consider the Cry65Aa1 to be a novel class of parasporin proteins.

To express Cry65Aa1, its coding gene was cloned into an *E. coli*-Bt shuttle expression vector, pBMB1A^23^, to generate the recombinant plasmid pBMB1A-65A and was then introduced into an acrystalliferous *B. thuringiensis* strain, BMB171. When sporulated cells were examined by phase-contrast microscopy, no crystalline inclusions were produced by BMB171/pBMB1A-65A (Fig. 1D, panel a). SDS-PAGE analysis also revealed no band corresponding to the predicted molecular weight of Cry65Aa1 (Fig. 1F, lane 1).

Why Cry65Aa1 was not expressed and did not form crystals is unclear. When analyzing the SBT-003 genome, we found ORF2, which was annotated as a hypothetical protein located downstream of *cry65Aa1*, and a typical terminator located downstream of *orf2* (Fig. 1C). Interestingly, sequence alignment revealed that ORF2 shared the highest level of identity with the C-terminal regions of Cry21Ba (46%), Cry21Fa (46%), Cry21Ga (45%), Cry12Aa (37%), and Cry5Ab (38%) (Fig. S5). With respect to *cry* operons, five reported cases (*cry10Aa*, *cry19Aa*, *cry30Ca, cry39Aa*, and *cry40Aa*) have gene organizations similar to the *cry65Aa* operon^24^,^25^. In these five similar operons, the upstream reading frames encode an N-terminal domain or a 65-kDa range Cry protein. However, Cry65Aa1 is a three-domain Cry protein and possesses N- and C- termini similar to the 130-kDa mass range Cry proteins (Fig. 1C).

We then cloned the entire operon into the vector pHT304 and generated the plasmid pBMB1331, which we introduced into BMB171 cells for expression. Phase-contrast (Fig. 1D, panel b) and electron (Fig. 1E) microscopy observations revealed that BMB171/pBMB1331 formed square-shaped parasporal crystals. SDS-PAGE analysis revealed that BMB171/pBMB1331 produced a major protein of 118 kDa in size (Fig. 1F, lane 2) that corresponds to the predicted molecular weight of Cry65Aa1.

### ORF2 is required for expression and crystallization of Cry65Aa1

**Figure 2:**
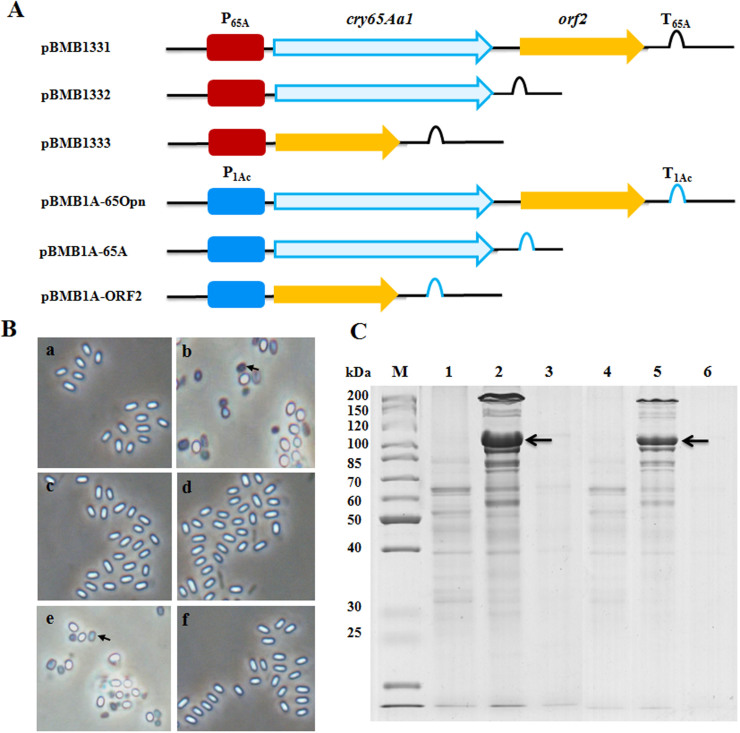
ORF2 is required for expression and crystallization of Cry65Aa1. (A), Schematic illustration of the different constructs containing *cry65Aa1* or *orf2*. (B), Phase-contrast micrographs of recombinant *B. thuringiensis* BMB171 strains. a, BMB171/pBMB1332; b, BMB171/pBMB1331; c, BMB171/pBMB1333; d, BMB171/pBMB1A-65A; e, BMB171/pBMB1A-65Opn; f, BMB171/pBMB1A-ORF2. Arrows indicate crystals. (C), SDS-PAGE analysis of the recombinant *B. thuringiensis* BMB171 strains. Lane M, protein molecular mass marker; lane 1, BMB171/pBMB1332; lane 2, BMB171/pBMB1331; lane 3, BMB171/pBMB1333; lane 4, BMB171/pBMB1A-65A; lane 5, BMB171/pBMB1A-65Opn; lane 6, BMB171/pBMB1A-ORF2. The position of Cry65Aa1 is indicated by black arrows.

This study is the first to include *orf2* downstream of the 130-kDa mass range Cry protein gene. To determine the function of ORF2 in this operon, a series of recombinant plasmids were constructed to analyze the expression and crystallization of Cry65Aa1 in BMB171 (Fig. 2A).

When sporulated cells were examined by phase-contrast microscopy, crystalline inclusions were produced only when both *cry65Aa1* and *orf2* were present as an operon in the constructs (Fig. 2B). The *B. thuringiensis* BMB171 strain that contained the entire native *cry65Aa1* operon (BMB171/pBMB1331 and BMB171/pBMB1A-65Opn) produced typical square-shaped crystals (Fig. 2B, panels b, and e). However, *B. thuringiensis* BMB171 strains containing only the intact *cry65Aa1* (BMB171/pBMB1332 and BMB171/pBMB1A-65A) or *orf2* alone (BMB171/pBMB1333 and BMB171/pBMB1A-ORF2) did not produce visible crystals or inclusions during sporulation (Fig. 2B, panels a, c, d, and f). The total protein profiles of BMB171/pBMB1331 and BMB171/pBMB1A-65Opn showed that both strains produced Cry65Aa1 (Fig. 2C, lane 1), which was consistent with phase-contrast microscopy observations. The protein profiles of the other acrystalliferous recombinant strains all lacked a distinct band corresponding to Cry65Aa1 (Fig. 2C). These results demonstrated that ORF2 is required for Cry65Aa1expression and crystallization.

### ORF2 has a stabilizing effect on *cry65Aa1* mRNA

**Figure 3:**
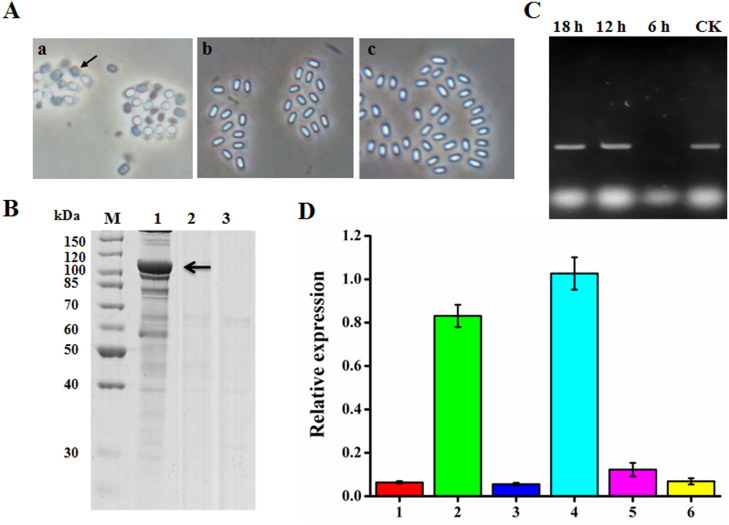
ORF2 has a stabilizing effect on *cry65Aa1* mRNA. (A), Phase-contrast micrographs of recombinant *B. thuringiensis* BMB171 strains. a, BMB171/pBMB1331; b, BMB171/pBMB1332+pBMB1A-ORF2; c, BMB171/pBMB1333+pBMB1A-65A. Arrows indicate crystals. (B), SDS-PAGE analysis of the recombinant *B. thuringiensis* BMB171 strains. Lane M, protein molecular mass marker; lane 1, BMB171/pBMB1331; lane 2, BMB171/pBMB1332+pBMB1A-ORF2; lane 3, BMB171/pBMB1333+pBMB1A-65A. The position of Cry65Aa1 is indicated by arrows. (C), the transcription profile of the non-coding region between *cry65Aa1 and orf2.* (D), the relative expression of *cry65Aa1* in different recombinant *B. thuringiensis* strains. 1, BMB171/pBMB1A-65A; 2, BMB171/pBMB1A-65Opn; 3, BMB171/pBMB1332; 4, BMB171/pBMB1331; 5, BMB171/pBMB1332+pBMB1A-ORF2; 6, BMB171/pBMB1333+pBMB1A-65A. In each case, the expression of *cry65Aa1* in BMB171/pBMB1331 had relative expression equal to 1.

The role that ORF2 played in Cry65Aa1 expression and crystallization was unclear. The results presented above demonstrated that the presence of *orf2* in *cis* can aid in *cry65Aa1* expression and crystallization. Therefore, we also examined *trans* expression of *cry65Aa1* and *orf2*. Interestingly, *B. thuringiensis* strains containing *trans* *cry65Aa1* and *orf2* in two different plasmids (BMB171/pBMB1332+pBMB1A-ORF2 and BMB171/pBMB1333+pBMB1A-65A) did not produce visible crystals or inclusions during sporulation (Fig. 3A, panels b and c). The SDS-PAGE results of the protein profiles of these two recombinant strains also both lacked distinct bands corresponding to Cry65Aa1 (Fig. 3B). These results demonstrate that the presence of *orf2* in *cis* is essential for Cry65Aa1 expression and crystallization, indicating that the *orf2* sequence may have a function at the *cry65Aa1* transcription level. Therefore, we believe that *cry65Aa1* and *orf2* may act as a single operon. To confirm this hypothesis, we designed a pair of primers (Coe-F and Coe-R) to amplify the region that spans the entire noncoding region between *cry65Aa1* and *orf2.* We analyzed the transcription pattern of this region at different growth stages of BMB171/pBMB1331 and found that this region can give rise to transcripts at 12 h and 18 h (Fig. 3C). These results demonstrated that *cry65Aa1* and *orf2* were co-transcribed under the control of the *cry65Aa1* promoter and are assembled as an operon.

We then assessed the transcript levels of *cry65Aa1* mRNA in the above strains at 12 h. The transcript profiles showed that BMB171/pBMB1331 and BMB171/pBMB1A-65Opn produced the highest levels of *cry65Aa1* mRNA (Fig. 3D), whereas the transcript profiles of other strains showed very low levels of *cry65Aa1* mRNA (Fig. 3D). These results were consistent with phase-contrast microscopy and SDS-PAGE analysis, which indicated that BMB171/pBMB1331 and BMB171/pBMB1A-65Opn formed crystals and produced the highest levels of Cry65Aa1 protein. These results indicate that ORF2 has a stabilizing effect on *cry65Aa1* mRNA and can promote *cry65Aa1* mRNA accumulation.

### *orf2* mediates its mRNA-stabilizing effect on Cry65Aa1 through secondary structure

**Figure 4:**
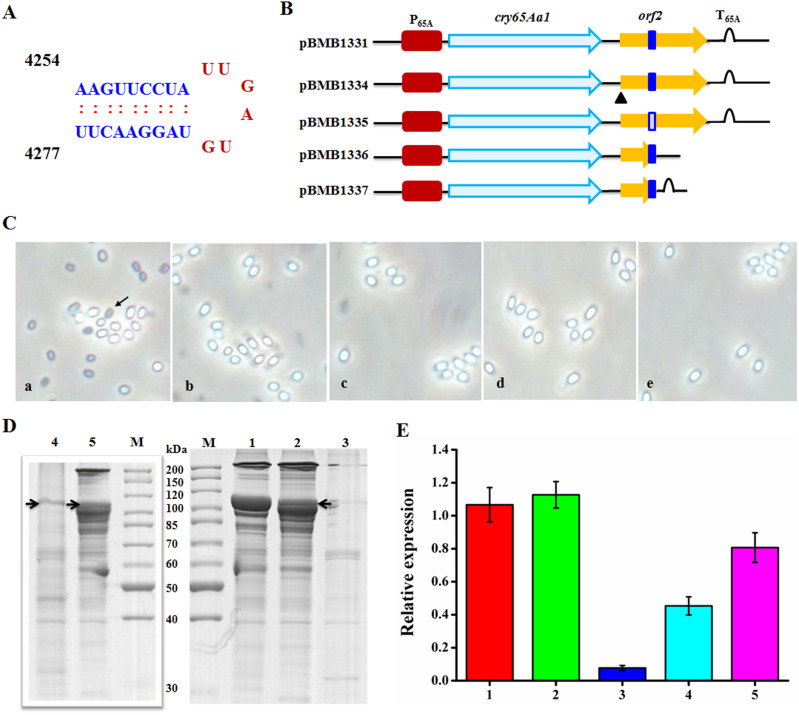
The stem-loop structure in *orf2* determined its mRNA stabilizing effect on Cry65Aa1. (A), the position and structure of the stem-and-loop structure in the internal of ORF2. (B), Schematic illustration of the different constructs containing *cry65Aa1* or *orf2*. (C), Phase-contrast micrographs of recombinant *B. thuringiensis* BMB171 strains. a, BMB171/pBMB1331; b, BMB171/pBMB1334; c, BMB171/pBMB1335; d, BMB171/pBMB1336; e, BMB171/pBMB1337. Arrows indicate crystals. (D), SDS-PAGE analysis of the total proteins produced by recombinant *B. thuringiensis* BMB171 strains. Lane M, protein molecular mass marker; lane 1, strain BMB171/pBMB1331; lane 2, strain BMB171/pBMB1334; lane 3, strain BMB171/pBMB1335; lane 4, strain BMB171/pBMB1336; lane 5, strain BMB171/pBMB1337. The position of Cry65Aa1 is indicated by black arrows. (E), the relative expression of *cry65Aa1* in different recombinant *B. thuringiensis* strains at 12 h. 1, BMB171/pBMB1331; 2, BMB171/pBMB1334; 3, BMB171/pBMB1335; 4, BMB171/pBMB1336; 5, BMB171/pBMB1337. In each case, the expression of *cry65Aa1* in BMB171/pBMB1331 had relative expression equal 1.

We provided evidence that ORF2 has a stabilizing effect on *cry65Aa1* mRNA. However, its role in Cry65Aa1 transcription was unclear. The results described above demonstrated that the presence of *orf2* in *cis* is essential for high level transcription of the *cry65Aa1* gene. However, when *orf2* is present in *trans* or is absent from the *cry65A* operon, the *cry65Aa1* gene cannot be expressed at normal levels. This observation indicated that the sequence of *orf2* but not the ORF2 protein may determine its mRNA-stabilizing effect. To confirm this hypothesis, we analyzed the DNA sequence of *orf2* and identified a typical stem-loop structure in *orf2* coding region (Fig. 4A). We then constructed a series of recombinant plasmids to analyze the functional implications of this structure on Cry65Aa1 transcription, synthesis and crystallization (Fig. 4B).

We examined sporulated *B. thuringiensis* BMB171 strains transfected with plasmids carrying one of four *orf2* mutants, by using phase-contrast microscopy. The first mutant plasmid was pBMB1334, in which *orf2* had a mutation from ATG to TAA at the original start site. This mutant *orf2* retained the stem-loop structure but could not express ORF2 protein. The second mutant was ‘broken *orf2*’: the plasmid pBMB1335 contained *orf2* with a mutation that removed the stem-loop structure from bases 4254–4277 of the operon. The third mutant was ‘intact *orf2*’: the plasmid pBMB1336 carried a truncated *orf2* that retained the region from ATG to base 4300. The fourth mutant was pBMB1337, which carried a truncated *orf2* that retained the region from ATG to base 4300 and the terminator of the Cry65A operon. None of these mutants produced visible crystals or inclusions during sporulation (Fig. 4C, panels b, c, and d). The protein profiles of BMB171/pBMB1331, BMB171/pBMB1334, and BMB171/pBMB1337 showed very high levels of Cry65Aa1 production (Fig. 4D, lanes 1, 2). In addition, BMB171/pBMB1334 and BMB171/pBMB1337 showed an extra major band at 100 kDa, which may correspond to Cry65Aa1, indicating that the Cry65Aa1 protein is being severely degraded (Fig. 4D, lane 2). The protein profiles of the recombinant strain BMB171/pBMB1336 showed very low levels of Cry65Aa1 (Fig. 4D, lane 4), whereas BMB171/pBMB1335 lacked a distinct band corresponding to Cry65Aa1 (Fig. 4D, lane 3).

To further analyze the function of the stem-loop structure on transcription, we analyzed the transcript levels of cry65Aa1 mRNA in the above strains at 12 h. We found that strains BMB171/pBMB1334 and BMB171/pBMB1331 produced similarly high levels of *cry65Aa1* mRNA. BMB171/pBMB1337 produced the next highest level of *cry65Aa1* mRNA. However, the transcript profile of BMB171/pBMB1335 indicated a very low level of *cry65Aa1* mRNA production (Fig. 4E). These results were consistent with SDS-PAGE analysis.

Summarize the above results, when there is no ORF2 protein but an *orf2* sequence is present (BMB171/pBMB1334), Cry65Aa1 can be transcribed and expressed at high levels. When the stem-loop structure is missing (BMB171/pBMB1335), Cry65Aa1 cannot be transcribed or expressed. When the stem-loop structure is retained, Cry65Aa1 can be transcribed and expressed, but at lower levels than in the presence of the wild type *orf2* (BMB171/pBMB1337). These results demonstrated that the stem-loop structure in *orf2* determined its mRNA-stabilizing effect on Cry65Aa1.

### The ORF2 protein functions as a C-terminal crystallization domain for Cry65Aa1

We also noted that, when retaining the stem-loop structure but not expressing the ORF2 protein, the recombinant strains were able to transcribe and express Cry65Aa1 but could not form visible crystals. This finding indicated that, in addition to functioning as an mRNA stabilizer, the ORF2 protein may have an important role in Cry65Aa1 crystallization.

**Figure 5:**
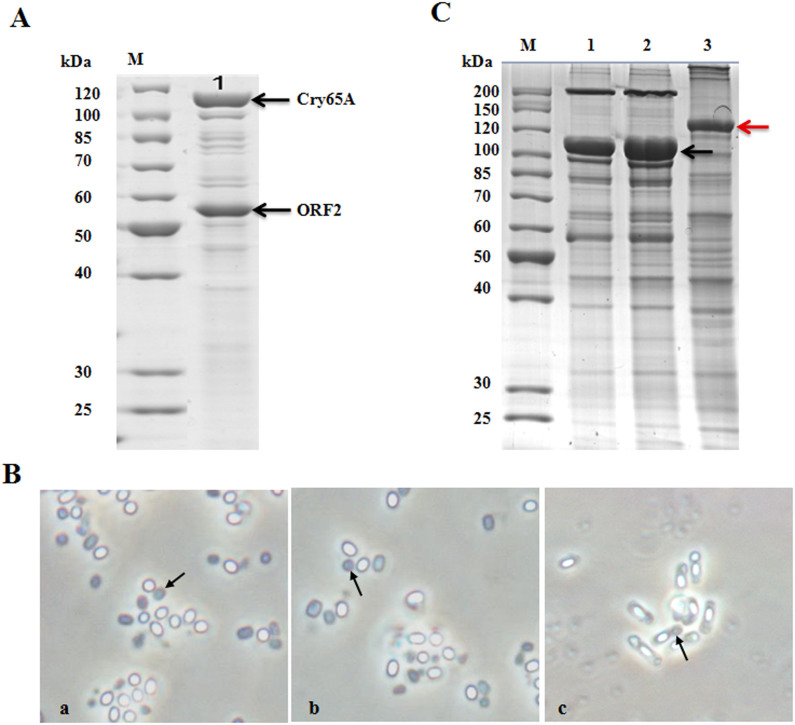
ORF2 functions as a C-terminal crystallization domain for Cry65Aa1. (A), SDS-PAGE analysis of purified Cry65Aa1 crystals from BMB171/pBMB1331. The position of Cry65Aa1 and ORF2 are indicated by arrows. (B), Phase-contrast micrographs of recombinant *B. thuringiensis* BMB171 strains. a, BMB171/pBMB1334+pBMB1A-ORF2; b, BMB171/pBMB1337+pBMB1A-ORF2; c, BMB171/pBMB21B-N::ORF2. Arrows indicate crystals. (C), SDS-PAGE analysis of the recombinant *B. thuringiensis* BMB171 strains. Lane M, protein molecular mass marker; lane 1, strain BMB171/pBMB1334+pBMB1A-ORF2; lane 2, strain BMB171/pBMB1337+pBMB1A-ORF2; lane 3, strain BMB171/pBMB21B-N::ORF2. The position of Cry65Aa1 and recombinant Cry21Ba are indicated by black arrow and red arrow, receptivity.

In the above results, *B. thuringiensis* BMB171 strains containing either intact *orf2* (BMB171/pBMB1334) or broken *orf2* (BMB171/pBMB1337) expressed the Cry65Aa1 protein but did not produce visible crystals (Fig. 4C, panels b, e). These two strains contained the *orf2 cis* factors but did not express ORF2. We considered the possibility that crystal assembly of Cry65Aa1 required ORF2 proteins. To test this hypothesis, we transformed these two recombinant strains with pBMB1A-ORF2 into to add ORF2 protein. Microscopy revealed that the recombinant strains BMB171/pBMB1334+pBMB1A-ORF2 (Fig. 5B, panel a) and BMB171/pBMB1337+pBMB1A-ORF2 (Fig. 5B, panel b) produced typical square-shaped crystals similar to those produced by BMB171/pBMB1331. Additionally, the SDS-PAGE profiles of these two strains revealed the presence of the predicted Cry65Aa1 bands (Fig. 5C, lanes 1, 2). These results indicated that the crystal assembly of Cry65Aa1 requires ORF2 proteins.

We then purified crystals from BMB171/pBMB1331. SDS-PAGE analysis showed that the purified crystals produce two major bands 118 and 58 kDa in size (Fig. 5A). The 118-kDa band corresponds to Cry65Aa1, and the 58-kDa band corresponds to the predicted molecular weight of ORF2. To confirm whether the 58-kDa band was ORF2, the band was excised from SDS-PAGE gels and examined by mass spectroscopy. A total of 80% of the peptides were encoded by ORF2, as identified from matrix-assisted laser desorption/ionization tandem mass spectrometry (MALDI-TOF/TOF-MS) results (Fig. S6). These results suggested that the ORF2 protein assembles with Cry65Aa1 forms crystals.

Sequence alignment revealed that ORF2 shares the highest level of similarity with the C-terminal regions of some reported Cry proteins (Fig. S5). Therefore, we hypothesized that ORF2 may function as a C-terminal domain to aid in Cry65Aa1 crystallization. To test this hypothesis, we constructed a recombinant plasmid (pBMB21B-N::ORF2) that contained the Cry21Ba N-terminus (Cry21Ba-N) and *orf2* in-frame. Using this construct, we analyzed whether ORF2 can help other crystal proteins to assemble into crystals, as the C-terminal domain does in 130-kDa crystal proteins. We chose Cry21Ba1 because ORF2 has the highest similarity to the C-terminal domain of Cry21Ba1. Microscopy of sporulated cells of the recombinant strain BMB171/pBMB21B-N::ORF2 (Fig. 5B, panel c) produced typical bipyramidal crystals, similar to those of the wild-type Cry21Ba in BMB171. Additionally, the SDS-PAGE profiles of sporulated cultures showed the presence of the predicted bands (Fig. 5C, lane 3). Taking these findings together, we concluded that ORF2 is required for the expression and crystallization of Cry65Aa1 and functions as a C-terminal crystallization domain.

### The crystallization of Cry65Aa1 requires two C-termini

**Figure 6:**
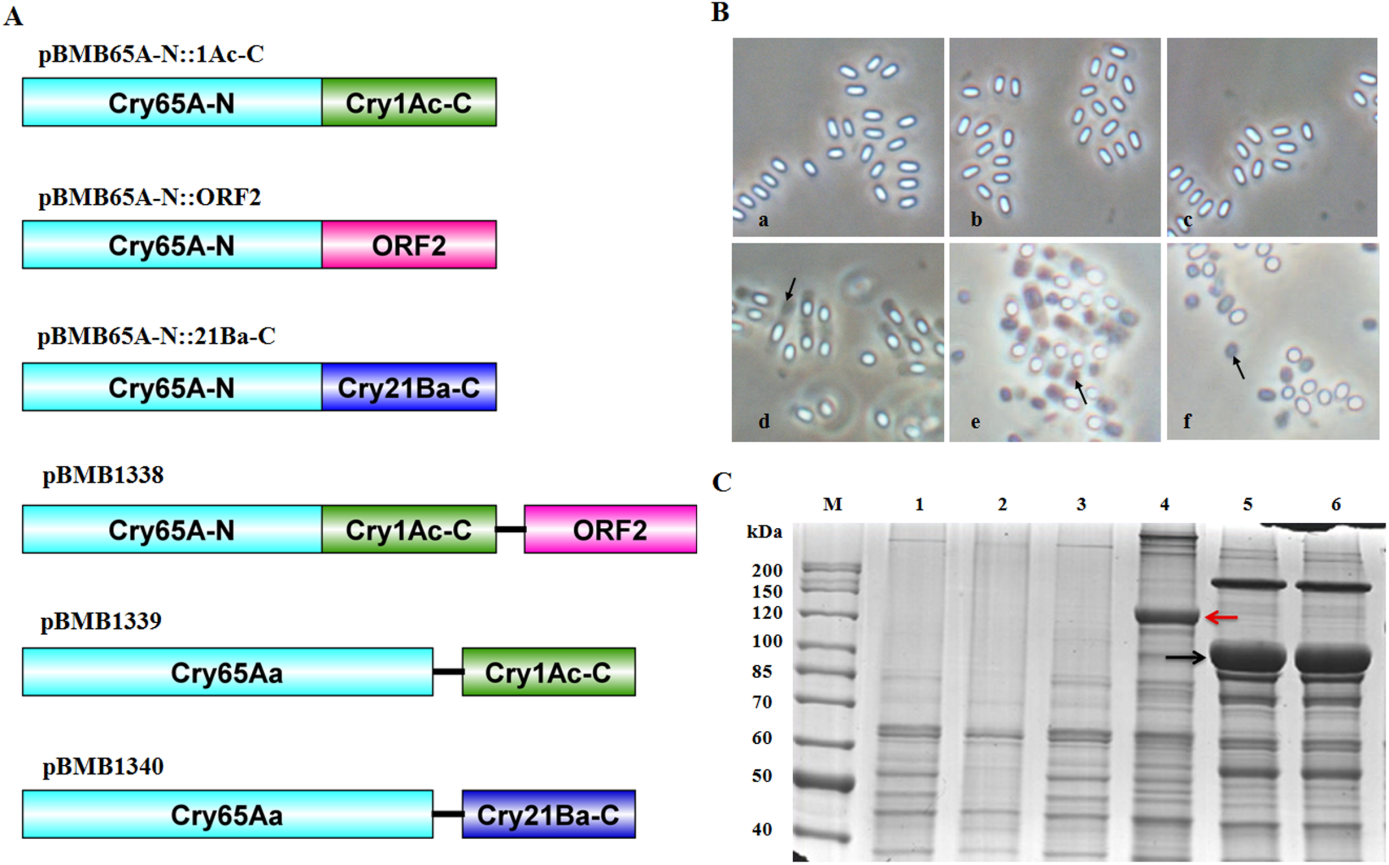
The crystallization of Cry65Aa1 requires two C-termini. (A), Schematic illustration of the different constructs containing *cry65Aa1* or *orf2*. (B), Phase-contrast micrographs of recombinant *B. thuringiensis* BMB171 strains. a, BMB171/pBMB65A-N::1Ac-C; b, BMB171/pBMB65A-N::ORF2; c, BMB171/pBMB65A-N:: 21Ba-C; d, BMB171/pBMB1338; e, BMB171/pBMB1339; f, BMB171/pBMB1340. Arrows indicate crystals. (C), SDS-PAGE analysis of the recombinant *B. thuringiensis* BMB171 strains. Lane M, protein molecular mass marker; lane 1, strain BMB171/pBMB65A-N::1Ac-C; lane 2, strain BMB171/pBMB65A-N::ORF2; lane 3, strain BMB171/pBMB65A-N::21Ba-C; lane 4, strain BMB171/pBMB1338; lane 5, strain BMB171/pBMB1339; lane 6, strain BMB171/pBMB1340. The position of Cry65Aa1 and recombinant Cry65A-N::1Ac-C is indicated by black arrow and red arrow, receptivity.

We have shown that intact *cry65Aa1* cannot be expressed or produce visible crystals under the control of either P65 or P1Ac (Fig. 1 and Fig. 2). Structural analysis revealed that the Cry65Aa1 C-terminal domain has 333 aa, which is shorter than the typical C-terminal domain (Fig. 1C). It is possible that the short form C-terminal domain is degenerate and cannot aid the N-terminal domain of Cry65Aa1 in expression and crystallization. To test this, we constructed 3 recombinant plasmids, as illustrated in Fig. 6A. The plasmid pBMB65A-N::1Ac-C contained the Cry65Aa1 N-terminus (Cry65A-N) and the C-terminal domain of Cry1Ac in-frame, and pBMB65A-N::21Ba-C contained the Cry65Aa1 N-terminus (Cry65A-N) and Cry21Ba in frame. These two constructs were used to analyze whether a typical C-terminal domain contributes to the crystallization of Cry65Aa1. In addition, pBMB65A-N::ORF2 contained the Cry65Aa1 N-terminus (Cry65A-N) and ORF2 in frame and was used to analyze whether ORF2 contributes to the crystallization of Cry65Aa1.

Microscopy of sporulated cells showed that none of these strains produced visible crystals or inclusions during sporulation (Fig. 6B, panels a–c). Additionally, the SDS-PAGE profiles of sporulated cultures showed that all analyzed recombinant strains lacked distinct bands corresponding to Cry65Aa1 (Fig. 6C, lanes 1–3). These results indicated that Cry65A-C, ORF2, or a typical C-terminal domain does not contribute to the expression or crystallization of Cry65Aa1 when fused in-frame, such as those in 130-kDa crystal proteins.

Owing to the complex nature of these results, we have summarized all of the former constructs that can form crystals (Table 1). We found that the recombinant strains that can form crystals all contain *cry65A* and *orf2* in *trans* or *cis*. This finding indicated that the crystallization of Cry65Aa1 might require two C-termini: the Cry65A C-terminal domain in addition to that of ORF2. To test this hypothesis, we constructed 3 additional recombinant plasmids: pBMB1338 (containing the Cry65A N-terminus [Cry65A-N] and the C-terminal domain of Cry1Ac in-frame and ORF2 in an operon), pBMB1339 (containing Cry65A and Cry1Ac-C in an operon), and pBMB1340 (containing Cry65A and Cry21Ba-C in an operon).

Microscopy of sporulated cells of the recombinant strains BMB171/pBMB1338 (Fig. 6B, panel c) showed typical bipyramidal crystals. Additionally, the SDS-PAGE profiles of sporulated cultures of BMB171/pBMB1338 showed the presence of the predicted bands (Fig. 6C, lane 4). Microscopy of sporulated cells of the strains BMB171/pBMB1339 and BMB171/pBMB1340 (Fig. 6B, panels d, f) identified typical square-shaped crystals similar to those produced by the wild-type operon in BMB171/pBMB1331. Additionally, the SDS-PAGE profiles of the sporulated cultures of these two recombinant strains showed the presence of the predicted Cry65Aa1 bands (Fig. 6C, lanes 5, 6).

Taking these findings together, we concluded that the crystallization of Cry65Aa1 requires two C-termini: its own C-terminal domain in addition to ORF2 or a typical C-terminal domain.

### Cry65Aa1 exhibits specific cytotoxicity toward MDA-MB231 cancer cells

**Figure 7:**
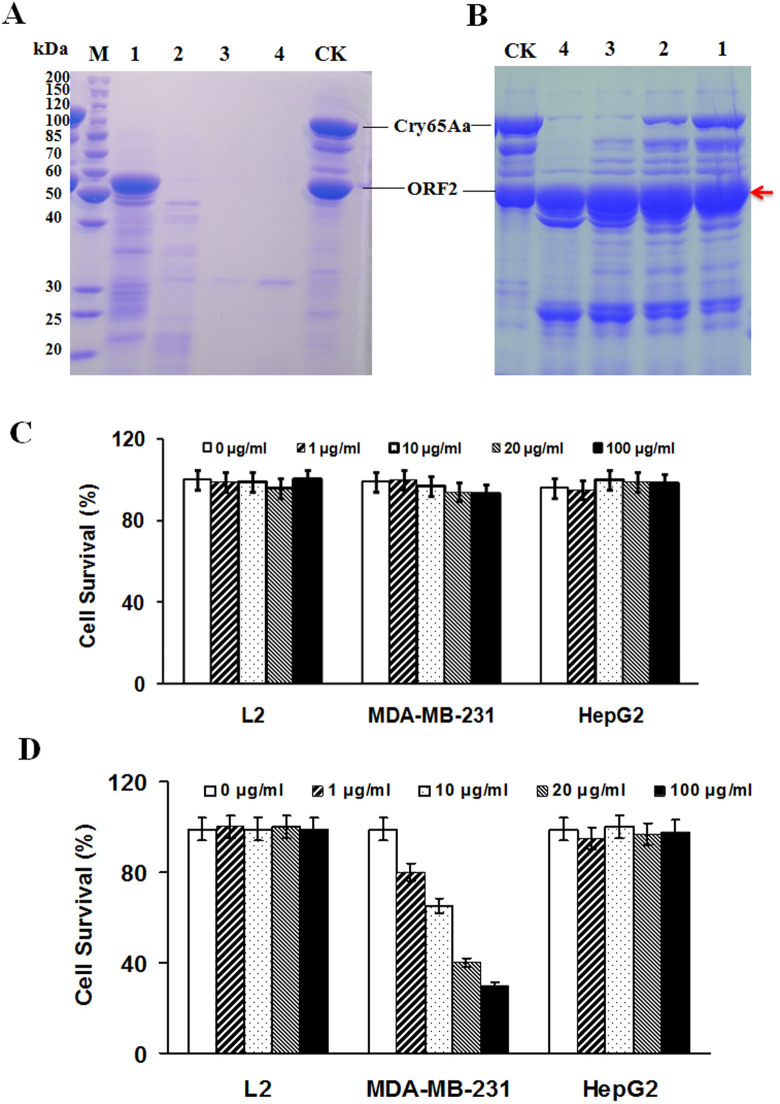
Cry65Aa1 exhibits specific cytotoxicity toward MDA-MB231 cancer cells. Solubilized Cry65Aa1 proteins were treated with proteinase K (A) and trypsin (B). Lane M, protein molecular mass marker; Lane Ck, 1, 2, 3, and 4 standards the samples after proteinase treated by final concentration 0, 1, 10, 100 and 200 μg/ml, respectively. Arrows showed the band of activated Cry65Aa1. The cytotoxicity of non-activated (C) and trypsin activated (D) Cry65Aa1 protein on different cell lines was measured by MTT method.

Sequence analysis indicated that Cry65Aa1 is a novel class of parasporin; thus, we examined the cytotoxic effect of Cry65Aa1 on cancer cell lines. It is known that some Cry proteins must be activated by proteinase before they exhibit activity; therefore, solubilized Cry65Aa1 proteins were treated with proteinase K and trypsin. We found that Cry65Aa1 can be activated by trypsin, as a 55-kDa stable band was observed (Fig. 7B), which is similar to that observed for other characterized Cry three-domain toxins. The cytotoxic results are shown in Fig. 7. We found that there was no cytotoxic effect of non-activated Cry65Aa1 on any tested cell line (Fig. 7C). Although activated Cry65Aa1 exhibits high cancer cell-killing activity toward MDA-MB231 cells, it has no cytotoxic effect on HepG2 and L2 cells. Additionally, MDA-MB231 cytotoxicity mediated by activated Cry65Aa1 exhibited a significant dose-dependent response (Fig. 7D). We suggest that the activated Cry65Aa1 protein has a selective cytotoxic effect on MDA-MB231 cancer cells.

To analyze whether the Cry65Aa1 protein possesses other properties, its effects on insect and nematode activities were determined, using solubilized and activated Cry65Aa1. Neither form of Cry65Aa1 had any killing activity toward the tested insect and nematode larvae.

## Discussion

Many parasporin proteins have been isolated from *B. thuringiensis*. These proteins have been divided into six types (PS1–6) by the Committee of Parasporin Classification and Nomenclature^26^. Here, we report a novel parasporin protein, Cry65Aa1, which has a selective cytotoxic effect on MDA-MB231 cancer cells (Fig. 7D). This novel parasporin protein may therefore be useful for the diagnosis and treatment of breast cancer. It also adds to the substantial number of identified parasporins and may be useful in comparative studies that aim to elucidate the mode of action of parasporins.

Since the employment of next-generation sequencing technology to discover new genes, the number of new *cry* genes has increased rapidly^21^. However, an increasing number of *cry* genes cannot be expressed when cloned^13^,^19^,^20^,^21^. Here, we found that Cry65Aa1 cannot be expressed when solely under its own control or the control of the Cry1Ac promoter (Fig. 1E and Fig. 2C). Interestingly, the *orf2* gene is located downstream of *cry65Aa1* (Fig. 1B). We found that ORF2 assisted with Cry65Aa1 expression (Fig. 2C, lanes 2, 5) and crystallization (Fig. 2B, panels b, e). Our result indicated that *cry65Aa1* cannot be expressed without ORF2. Because this observation may apply to other *cry* genes, the Cry65Aa1 expression system provides an appropriate strategy to promote the expression and crystallization in other *cry* genes.

Five reported *cry* genes (*cry10Aa*, *cry19Aa*, *cry30Ca, cry39Aa*, and *cry40Aa*) have gene organizations similar to the *cry65Aa* operon^24^,^25^ (Fig. 1C). In particular, Barboza-Corona et al^25^ demonstrated that ORF2 could enhance the synthesis and crystallization of Cry19A by functioning as a C-terminal crystallization domain. Our results also demonstrate that ORF2 in the *cry65Aa* operon has a C-terminal crystallization domain function and promotes Cry65Aa1 crystallization. However, there are differences between the *cry65Aa* and *cry19A* operons. First, we found that the *orf2* sequence of the *cry65A* operon has an mRNA-stabilizing effect function on *cry65Aa* mRNA (Fig. 3). Furthermore, we showed that there is a typical stem-loop structure in the *orf2* coding region and that this structure affects the mRNA-stabilizing effect on *cry65Aa* mRNA (Fig. 4). Most likely, the translation of ORF2 prevents the stem-loop formation that results in mRNA stability, instead of ORF2 having a direct effect on mRNA stabilizing activity. The mechanisms that can improve Cry protein expression in *B. thuringiensis* have been studied at the transcriptional, posttranscriptional, and posttranslational levels. Factors containing promoters^27^, stable mRNA factors (such as STAB-SD sequences^28^), and accessory proteins (such as P19 or P20^29^), have been identified. Our results provide a novel understanding of the function of ORF2. We also reveal a novel mechanism that can improve Cry protein expression in *B. thuringiensis.*

In addition, the upstream reading frames of five similar operons all encode an N-terminal domain or a 65-kDa-range Cry protein (Fig. 1C). For the *cry19A* operon, it was speculated that mutations that accrued in the extant intergenic region between *cry19A* and *orf2* resulted in this two-gene operon and that the Cry19A (75 kDa) and ORF2 (60 kDa) proteins together have features that are similar to those of 135-kDa Cry proteins^25^. However, Cry65Aa1 is a three-domain Cry protein and possesses N- and C- termini similar to the 130-kDa mass range Cry proteins. It is known that most Cry proteins that have a mass in the 130-kDa range typically do not require other proteins for synthesis and crystallization. Thus, the genes that encode these proteins occur alone rather than in operons^1^. However, the *cry65A* operon includes an *orf2* downstream of the Cry proteins in the 130-kDa mass range. We do not have a clear explanation for why the 130-kDa mass range Cry65Aa1 exists as a two-gene operon that also encodes the crystallization domain-like ORF2. The C-terminal domain of Cry65Aa1 is shorter than the typical C-terminal domain (Fig. 1A). Given that Cry65Aa1 did not crystallize in the presence of its own C-terminal domain alone (Fig. 6), it is tempting to speculate that some mutations have accrued in its C-terminus and have resulted in C-terminal degradation and the partial loss of the capacity to form crystals. Thus, the protein requires an additional C-terminal domain to complement its own crystallization capacity. However, when the sequence was analyzed, we found that the Cry65Aa1 C-terminal domain has low homology with a hypothetical protein from *V. ratti* ACS-216-V-Col6b, but not with any reported C-terminal domains (Fig. S4). Consequently, another possibility is that, earlier in evolution, Cry65Aa1 was an N-terminal domain or a 65-kDa-range Cry protein member. In the evolutionary process, the N-terminal domain may have fused with other proteins to produce the novel protein Cry65Aa1. However, at the synthesis and crystallization levels, this novel protein still requires the help of a C-terminal domain such as ORF2, which accounts for the current composition of the operon.

The ORF2 in the *cry65A* operon has highest homology with the C-terminal domain of some nematicidal Cry proteins (Fig. S5), but Cry65Aa1 has highest homology with a parasporin, Cry41Aa (Fig. S4). We found that Cry65Aa1 has a selective cytotoxic effect on MDA-MB231 cancer cells (Fig. 7) but no anti-nematode activity. These findings suggested that Cry65Aa1 and ORF2 might have originated from different *B. thuringiensis* strains that had different ecological niches. Here, we suggest that the coexistence of *cry65Aa1* and *orf2* in SBT-003 is the result of a long evolutionary process. It is possible that *cry65Aa1* and *orf2* may exist separately in some strains that cannot express and form crystals and therefore have previously been overlooked because of a *B. thuringiensis* strain-screening strategy that is based on crystal formation. The downstream *orf2* arrangement is not unique; rather, it is present in several genes^24^,^25^. We are unaware of the exact significance of this arrangement in the Cry protein family. However, we envisage that this configuration may be an evolutionary strategy of some groups of Cry proteins, such as Cry65Aa1. That these groups of proteins cannot be expressed or form crystals in their mother cells may be due to the loss of ORF2 or some other similar factor. Furthermore, the existence of strains that contain *cry* genes but do not form crystals creates challenges for the phenotype-based *B. cereus* and *B. thuringiensis* classification system^30^,^31^; using the existing strategy, some *B. thuringiensis* strains containing *cry* genes with “no crystals” phenotype may be classified as *B. cereus*.

The discovery and application of novel crystal protein genes is considered to be an important approach to controlling and overcoming the potential resistance of certain pests. As a result, a growing number of *cry* genes have been identified using various strategies^4^,^11^. To discover novel *cry* genes, the typical starting point is to screen a novel *B. thuringiensis* strain. Thus far, a practical method of screening *B. thuringiensis* strains is to detect parasporal crystals by microscopy^30^,^31^. Using this method, a large number of *B. thuringiensis* strains have been isolated and maintained after detection of the parasporal crystals. All of the reported *cry* genes were cloned from *B. thuringiensis* strains that can form parasporal crystals. The Cry65A phenotype indicates that many “no crystal” *B. thuringiensis* strains that harbor novel *cry* genes may have been overlooked. The presence of these “no crystal” *cry* genes presents challenges to the new *cry* gene discovery strategy, which is based on either assessing crystals or SDS-PAGE profiles^17^,^18^,^21^. It can be envisaged that these overlooked *B. thuringiensis* strains contain a wealth of undiscovered *cry* genes. This situation has not been previously reported and requires future consideration when searching for novel *cry* genes. Our discovery provides a potential framework for isolating novel crystal protein genes from “no crystal” *B. thuringiensis* strains.

## Methods

### Bacterial strains, plasmids, and medias

The strains and plasmids used in this study are listed in Table S1. *Escherichia coli* and *B. thuringiensis* strains were maintained on Luria-Bertani (LB) medium and supplemented with appropriate antibiotics at 37°C and 28°C, respectively. For crystal protein preparation, *B. thuringiensis* strains were grown in ICPM liquid medium at 28°C until cell lysis. The transformation of *B. thuringiensis* was performed by electroporation, as described previously^32^.

### Crystal protein preparation, solubilization, activation and quantification

Crystal preparation, solubilization, and quantification were performed as previously described^33^. Solubilized Cry65Aa1 proteins were treated with proteinase K and trypsin (final concentrations: 0, 1, 10, 100 and 200 μg/ml) at 37°C for 90 min. The samples were then dialyzed overnight against 50 mM Tris (pH 9.0) at 4°C for subsequent bioassay.

### Sequence analysis

DNA sequences were determined, and primers were synthesized by AuGCT Biotechnology Co., Ltd (AuGCT, Beijing). Amino acid sequence alignments and phylogenetic trees were produced using MEGA 4.1 software. Analyses of primary and secondary protein structure predictions were performed using PHD software (http://www.predictprotein.org/). The protein sequences were compared to other proteins using BLAST.

### Microscopy

The sporulating cultures were monitored using an optical microscope with an oil immersion lens. For transmission electron microscopy observations, samples were treated using methods described previously^33^ and were examined using a Hitachi 7000 FA electron (Hitachi, Japan) microscope.

### Construction of recombinant plasmids

The primers used in this study are listed in Table S2, and the plasmid construction strategy is illustrated in Figures. 2A, 4B, and 6A.

The *cry65A* gene was amplified with primers 65A–F and 65A–R, and a 3.2-kb DNA fragment was then cloned into the *Bam*HI*-Xh*oI site of pUC18-T to generate the plasmid pEMB1330. Then, the 3.2-kb *cry65A* DNA fragment was cloned into the *Bam*HI*-Xh*oI site of the *E. coli*-Bt shuttle expression vector pBMB1A to generate pBMB1A-65A for expression. To clone the entire Cry65A operon, primers 65Opn-F and 65Opn-R were designed based on the SBT-003 genome sequence, for amplifying *cry65Aa1* together with *orf2* and its promoter and terminator. Then, a 5.3-kb PCR fragment was purified and cloned into the *Bam*HI*-Hind*III site of the *E. coli*-Bt shuttle vector pHT304 to generate pBMB1331. The primers 65A–F and ORF2-R-1 were used to amplify the *cry65Aa1* and *orf2* region, and the 4.7-kb PCR fragment was then digested with *Bam*HI*-Xh*oI and ligated into pBMB1A to generate pBMB1A-65Opn. The primers 65Opn-F and 65A-R were used to amplify the 3.5-kb putative promoter region of the *cry65A* operon and *cry65Aa1 orf* region. The primers T65-F and 65Opn-R were used to amplify the 0.2-kb terminator region of the *cry65A* operon. Then, the 3.5-kb and 0.2-kb DNA fragments were digested with *Xho*I and ligated together, and the products were digested with *Bam*HI*-Hind*III and ligated into pHT304 to generate pBMB1332. The primers ORF2-F-1 and ORF2-R-1 were used to amplify *orf2*, and the 1.5-kb PCR fragment was then digested with *Bam*HI*-Hind*III and ligated into pBMB1A to generate pBMB1A-ORF2. The primers 65Opn-F and P65-R (splice overlap extension [SOE] primers contain *orf2* 1–20-bp regions) were used to amplify the 0.4-kb putative promoter region of the *cry65A* operon. The primers ORF2-F-2 (SOE primers containing 20-bp regions of putative promoter regions) and 65Opn-R were used to amplify the 1.7 kb *orf2* and the terminator region of the *cry65A* operon. Then, the 0.4-kb and 1.7-kb DNA fragments were ligated by SOE PCR, and the products were digested with *Bam*HI*-Hind*III and ligated into pHT304 to generate pBMB1333.

### Construction of ORF2 mutants

The primers Morf2-1 and Morf2-2 were used to construct the ORF2 mutant by SOE with primers 65Opn-F and 65Opn-R, which changed the start codon ATG to the stop codon TAA. Then, the products were digested with *Bam*HI*-Hind*III and ligated into pHT304 to generate pBMB1334. The primers 65Opn-F and Morf2–3 were used to amplify the 1–4254 site fragment of the *cry65A* operon. The primers Morf2-4 and 65Opn-R were used to amplify the 4277–5366 bp fragment of the *cry65A* operon. Then, the 4.2-kb and 1.1-kb DNA fragments were digested with *Xh*oI and ligated together, and the products were digested with *Bam*HI*-Hind*III and ligated into pHT304 to generate pBMB1335, which removed the stem-loop structure of the *cry65A* operon. The primers 65Opn-F and Morf2–5 were used to amplify the 1–4300 bp fragment of the *cry65A*. Then, the 4.3-kb DNA fragment was digested with *Bam*HI*-Xh*oI and ligated into pHT304 to generate pBMB1336. The primers T65-F and 65Opn-R were used to amplify the 0.2-kb terminator region of the *cry65A* operon. Then, the 0.2-kb DNA fragment was digested with *Xh*oI*-Hind*III and ligated into pBMB1336 to generate pBMB1337.

### Chimeric protein construction

The C-terminal region of Cry1Ac contains 567 aa (from 612 to 1178), and the C-terminal region of Cry21Ba contains 606 aa (from 680 to 1286). The N-terminal region of Cry65Aa1 contains 731 aa (from 1 to 731), and the N-terminal region of Cry21Ba contains 680 aa (from 1 to 680). The primers 65Opn-F and 65A-N-R were used to amplify a 2.6-kb DNA fragment containing the putative promoter region of the *cry65A* operon and the N-terminal coding region of Cry65Aa1. The primers 1Ac-C-F and 1Ac-C-R were used to amplify a 1.9-kb DNA fragment containing the C-terminal coding region and the terminator of Cry1Ac. Then, the 2.6-kb and 1.9-kb DNA fragments were digested with *Sal*I and ligated together, and the products were digested with *Bam*HI*-Hind*III and ligated into pHT304 to generate pBMB65A-N::1Ac-C. Similarly, the promoters of Cry21Ba and Cry65A and their N-terminal half coding regions were amplified using the primers 21Ba-N-F, 21Ba-N-R, 65A-F and 65AN-R, respectively. Additionally, the coding regions of ORF2, C-terminal half of Cry21Ba, and their terminators were amplified using the primers ORF2-F-3, 65Opn-R, 21Ba-C-F and 21Ba-C-R, respectively. Then, the PCR DNA fragments were digested with *Sal*I and ligated together, and the products were digested with *Bam*HI*-Hind*III and ligated into pHT304 to generate pBMB21B-N::ORF2, pBMB65A-N::ORF2, and pBMB65A-N::21Ba-C. To test the hypothesis that the crystallization of Cry65Aa1 requires two C-termini, we constructed 3 other recombinant plasmids: pBMB1338 (containing the Cry65A N-terminus [Cry65A-N] and the C-terminal domain of Cry1Ac in-frame and *orf2* in an operon), pBMB1339 (containing Cry65A and Cry1Ac-C in an operon) and pBMB1340 (containing Cry65A and Cry21Ba-C coding regions in an operon).

All PCR products were purified using an OMEGA D2500-01 gel extraction kit (OMEGA, Shanghai), and recombinant plasmids were transformed in *E. coli* DH5α cells. The fragment integrity of all constructed plasmids was confirmed by restriction enzyme digestion and sequencing analysis (AuGCT, Beijing).

### Peptide identification

The peptides corresponding to SDS-PAGE bands were examined using MALDI-TOF/TOF-MS system (4700 Proteomics Analyzer, Applied Biosystems). Database searching of spectral data was conducted using the MASCOT search engine (www.matrixscience.com, Matrix Science).

### RNA extraction and expression analyses

Total RNA was extracted from *B. thuringiensis* strains using a total RNA isolation system (Promega, Madison, WI, USA). Analyses of *cry65Aa1* gene expression were performed using qPCR with the primers Qrt-F and Qrt-R; the 16s RNA gene was amplified as a quantitative control. The qPCR was conducted using IQ SYBR Green Supermix (BioRad, Hercules, CA, USA) according to the manufacturer's instructions. At least three independent biological samples were used for each individual strain. The data were normalized by the value of 16s RNA signal, and fold changes in expression levels were calculated and compared with those of strain pBMB1331/BMB171.

### Insect and nematode bioassays

The bioassays of toxicity toward insects, including *Plagiodera versicolora*, *Tribolium castaneum*, *Helicoverpa armigera*, *Plutella xylostella*, and *Bombyx mori* larvae, were conducted using methods described previously^33^. The nematode activity assays against *Caenorhabditis elegans* and *Meloidogyne hapla* were conducted using methods described previously^17^.

### Cytotoxicity assays

The cytotoxic effects of solubilized and activated Cry65Aa1 were tested on MDA-MB-231 cells, HepG2 cancer cell lines, and L2 cells by MTT assay^34^. The cell lines were obtained from CCTCC (Wuhan) and were maintained in RPMI 1640 medium (Gibco). Some of the other cells were in DMEM medium (Gibco) supplemented with 10% fetal bovine serum (FBS), 100 units/ml penicillin, and 100 μg/ml streptomycin in 5% CO_2_ at 37°C. Activated and non-activated Cry65Aa1 proteins were added to cultured mammalian cells at different concentrations (0, 1, 10, 20, 100 μg/ml). After preincubation at 37°C, cell proliferation was measured 24 h after administration.

## Author Contributions

M.S. and D.H.P. designed the study and wrote the manuscript. D.H.P., C.Y.P., H.W. and Q.H. participated in experiments, analyzed results, and contributed to the Methods/Figures. J.S.Z. performed the bioinformatics analysis. All authors have read and approved the final manuscript.

## Supplementary Material

Supplementary Information Supplementary Dataset 8

## Figures and Tables

**Table 1:** The summary of constructions which can express and form crystals

Strains	Genotype	Expression	Crystallization	Transcription
BMB171/pBMB1331	*cry65Aopn*	+++	+	+++
BMB171/pBMB1332	*cry65A*	-	-	-
BMB171/pBMB1333	*orf2*	-	-	/
BMB171/pBMB1A-65Opn	*cry65Aopn*	+++	+	+++
BMB171/pBMB1A-65A	*cry65A*	-	-	-
BMB171/pBMB1A-ORF2	*orf2*	-	-	/
BMB171/pBMB1332+pBMB1A-ORF2	*cry65A+ orf2, trans-*	-	-	-
BMB171/pBMB1332+pBMB1A-65A	*cry65A+ orf2, trans-*	-	-	-
BMB171/pBMB1334	*cry65A-*mutant *orf2*	++	-	++
BMB171/pBMB1335	*cry65A-*broken *orf2*	-	-	-
BMB171/pBMB1336	*cry65A-*intact *orf2*	+	-	+
BMB171/pBMB1337	*cry65A-*intact *orf2*	++	-	++
BMB171/pBMB1334+pBMB1A-ORF2	*cry65A-*mutant *orf2*+*orf2*	+++	+	/
BMB171/pBMB1337+pBMB1A-ORF2	*cry65A-*intact *orf2*+*orf2*	+++	+	/
BMB171/pBMB21B-N::ORF2	*cry21B-N::orf2*	+++	+	/
BMB171/pBMB65A-N::1Ac-C	*cry65A-N::1Ac-C*	-	-	/
BMB171/pBMB65A-N:: ORF2	*cry65A-N::orf2*	-	-	/
BMB171/pBMB65A-N:: 21Ba-C	*cry65A-N::21Ba-C*	-	-	/
BMB171/pBMB1338	*cry65A-N::1Aa-C-orf2*	+++	+	/
BMB171/pBMB1339	*cry65A-cry1Ac-C*	+++	+	/
BMB171/pBMB1340	*cry65A-cry21Ba-C*	+++	+	/

Note: +, representative the expression or transcription lever were below the 10% as that of wild type *cry65A* operon; ++, representative the expression or transcription lever were locate 10% to 50% as that of wild type *cry65A* operon; +++, representative the expression or transcription lever were similar as that of wild type *cry65A* operon;-, representative it has no expression or transcription products;/, representative not detected.
