# Supplementary material for: The expression and crystallization of Cry65Aa require two C-termini, revealing a novel evolutionary strategy of Bacillus thuringiensis Cry proteins
Source: Sci Rep. 2015 Feb 6;5:8291. doi: 10.1038/srep08291 (PMC4319155; doi:10.1038/srep08291)
Supplement: Supplementary Information — Supplementary Dataset 8 [file srep08291-s1.doc]

**Supplementary information:**

**The expression and crystallization of Cry65Aa require two C-termini, revealing a novel evolutionary strategy of *Bacillus thuringiensis* Cry proteins**

**Dong-hai Peng, Cui-yun Pang, Han Wu, Qiong Huang, Jin-shui Zheng, and Ming Sun^*^**

**Figures:**

1 CACTGGAACA AAAGAGTCAT CTGCAAAGAC AATAGACAGC TCTTTTGTAC CAAGTGTGTA TTATAAAATA TCTAGAGAAG ATGCTGATGC AATGGGATTA TATTAAATAG CTATGTATGA GCAAAGAAGA

131 TGATTCACAA TCTTTTTAAC ATGATTTAGA TTTTGTCGAT TACCTTGTTC TACATTTATG CATATGGCAT TACAAATTGC TTTTTACCAA AGGTGATTCA TAAGTATTTC ATCTATAGAT AAGAATGTAG

261 TTATCTTATA AATGCGTTAC TACTATCCAC TGTTTTTCTG AATTGTATAT GCTTTTTCCA TAGGTACACT TGCACATTTT TTTACAAATT GGTTCAAAAT ACCAAAAAGG GAGCATACAC TATTTTTGTG

**BtⅡ-10**

**BtⅠ-35**

**BtⅡ-35**

391 CTTACAATTA CTTTTAA**GGA GG**AATTATT**T TG**GCAGATAA TAATTTACAG TATACACCAC CACTAAGATT AGATGCTGAA GAATATGTAC CTTGGAAAGA TGTAGATTCT GATGTCATTA TTGACGCTGT

>>...................................................Cry65....................................................>

**RBS**

L A D N N L Q Y T P P L R L D A E E Y V P W K D V D S D V I I D A

521 AGCTGGAGCT GAAGGAGCCC TTTTCAATCT ATTAATTCCA GGCTCAGGTA AAATTGTAGA ATGGGGAGTA AGTAAGTTTT TAAAATGGGC TACTGGTGAC ACATATAAGC CTCCTAGTTT ATCACAAGAA

>...................................................................Cry65....................................................................>

V A G A E G A L F N L L I P G S G K I V E W G V S K F L K W A T G D T Y K P P S L S Q E

651 ATTACAAGAG TTGAAGGAAT GATCGCAACA CTACAACAAC AAATGTATAA TGTAATTAGC GCTACGAAAG CTGAAATTTT ACAAAATGTA GCAGGCTTAT TTTTCTCGAA CGTAGTAAGC GCTGTAAATG

>...................................................................Cry65....................................................................>

I T R V E G M I A T L Q Q Q M Y N V I S A T K A E I L Q N V A G L F F S N V V S A V N

781 ATTATCACCA TTACTTAGAG CGATGGCTAG ACAACTCTGA TGATTCCAAT AGGCTAGCAG AATTAAAAAG CTCTCTAGAT AAGGCAATTG ATGAAGCTCA TAAAGCTGTA TCAGCTTCGT ATTACAACCA

>...................................................................Cry65....................................................................>

D Y H H Y L E R W L D N S D D S N R L A E L K S S L D K A I D E A H K A V S A S Y Y N

911 AAATGCACAA TATACATTCG CATATAATGT GAATGCAGCT ACATTTTCCC TAATCCTGAT GCGAGATAAA TATTTGAATT ACACCCAGTG GGGCTATGGT GATGCAGCTG GTGCTCAAAA TTTTTATGAG

>...................................................................Cry65....................................................................>

Q N A Q Y T F A Y N V N A A T F S L I L M R D K Y L N Y T Q W G Y G D A A G A Q N F Y E

1041 GGCACATTCC TATACCGCTT AAAAGACTAT ACAACAAACA TTCTCACACA ATATAACGCC ATATTAAAAC TCGTACATGA CTTAGCAGTT AATCCGTATA GTGAGCCATA TAGAGAGGTT ACCAATCGAA

>...................................................................Cry65....................................................................>

G T F L Y R L K D Y T T N I L T Q Y N A I L K L V H D L A V N P Y S E P Y R E V T N R

1171 ATCCACTCCA GGTGACAACT AACCCTCTAT ATAATGGTAA GTCTGATCAA CGTAGCATGT ATTGGCCTTC TGCTTATTAC GGAGGTTCCG ACAACATGCG AAAAAAATGT ATCTCTCCCA TGGCTTCAGA

>...................................................................Cry65....................................................................>

N P L Q V T T N P L Y N G K S D Q R S M Y W P S A Y Y G G S D N M R K K C I S P M A S

1301 TCCATATAAA CACTTTTCAG ACTCTCGTAT AGAGAATCAA TGGAATGATG ACCAGCACCG CTCCTGTCAA ACACCACTTC CTAATGGCGG TTTAGAAATG CAGATTAATA TGCCAGATCA GTTAAAGCTC

>...................................................................Cry65....................................................................>

D P Y K H F S D S R I E N Q W N D D Q H R S C Q T P L P N G G L E M Q I N M P D Q L K L

1431 TCTTTCCAAT GGAATGCTTA CAATCGAACA CGCAATGTAT TAACTCAAAC TGGTTTAGAT TTTATAGCCA TTTGGCCTTA TTTTGATCCT ATACAATATC CACCAGGTGA AGTAACCGCA GATTTAACTC

>...................................................................Cry65....................................................................>

S F Q W N A Y N R T R N V L T Q T G L D F I A I W P Y F D P I Q Y P P G E V T A D L T

1561 GTATGCTTTA TTCCGATTTA GCCGGCGCTA TAACAAAAAA TGATATTCAA AGTGTGGATG ATATTGATAA TCACGTAAAA AAACAATCCG ATTTATTCGA ATTCTTGAAA AGCTCTAAAC TGTATACGAA

>...................................................................Cry65....................................................................>

R M L Y S D L A G A I T K N D I Q S V D D I D N H V K K Q S D L F E F L K S S K L Y T

1691 GAACGTTAAA GTACAAACTC AACAAGGCCC TCCATTTGCT TATGACTCAA GTACAGTCAA GACCCACGTA GATGGAGATA TTATTGTAGG AATTGAAAAT ACTACAGAAC GAACTTTAGA AACAAACACA

>...................................................................Cry65....................................................................>

K N V K V Q T Q Q G P P F A Y D S S T V K T H V D G D I I V G I E N T T E R T L E T N T

1821 CGCAATCCTG GAGGAATACA AGGAAGCAGT TCTGATAACG CTGATACTAT AGATCCTACA GAAGCATCTA AATCCGTTTC TATTTCCCAA TGGCTATTCC CACGCATTTT CAACTTTAAC GGCAAAGTAA

>...................................................................Cry65....................................................................>

R N P G G I Q G S S S D N A D T I D P T E A S K S V S I S Q W L F P R I F N F N G K V

1951 TTGGAAGTGT TAACGAAACG ACGCCAGGTA ATGTGACAGG AATTTATAAC AATTTAACCT ACGATTTCAC ATTACCAGAG CATCAAATTT CTTGGTTAAG CTATGTGCAA GCACAAGAAA ATGTATTAAG

>...................................................................Cry65....................................................................>

I G S V N E T T P G N V T G I Y N N L T Y D F T L P E H Q I S W L S Y V Q A Q E N V L

2081 GCCTACTAAT GGATACGACT ATCTAACAGG TAATTTAACC GCAGCAAACC AGGGGCAAAT TGGCGCTTTA GCCATTGGTT GGACAAAAGC AAATATTGAT GCAAATAATA CTGTAAAAAA AGGTACCATT

>...................................................................Cry65....................................................................>

R P T N G Y D Y L T G N L T A A N Q G Q I G A L A I G W T K A N I D A N N T V K K G T I

2211 ACCTCCATTC CGGCTGTTAA ATCTCATAGC ATCACAGATT GGGGTACAGG AACTGTTGTA AAAGGTCCTG GCCATACAGG TGGGGATTTA GTAAAATTAC CACCAAACAC TCGCGCAAAG ATGATTGTAA

>...................................................................Cry65....................................................................>

T S I P A V K S H S I T D W G T G T V V K G P G H T G G D L V K L P P N T R A K M I V

2341 CTATTGAGGA TACTTCAACT TCTTATGATG TTCGCATTCG TTATGCTGCA CCAAGTGGTG GACATATACA ATTCTCCTAT TGGAACGGTG GTAGCGATGT GAAGGTGGCA GATACTCAAT TACAAAATAC

>...................................................................Cry65....................................................................>

T I E D T S T S Y D V R I R Y A A P S G G H I Q F S Y W N G G S D V K V A D T Q L Q N

2471 AGGTGGGAAT ACTAATTTTG AACATTTTCA ATATGCAATG TTAACAACTG ACAACGCAAA ATTCAAAGCA CCGTTTTCAC CTGTAGAAAT ATTGATTGAG AATATTGGGG ATAGTGATGT GTACCTGGAT

>...................................................................Cry65....................................................................>

T G G N T N F E H F Q Y A M L T T D N A K F K A P F S P V E I L I E N I G D S D V Y L D

2601 AAAGTGGAGT TTTCAGTTCT TGGCCAACTC CCACCAGGAG CTACTCCACT CACATCTCAG TATATGAATC CTTATGGATA TAATATTCTT TGGCAATCAA AAAATGGGGA AGCGGCAAAT CAAGGTATGG

>...................................................................Cry65....................................................................>

K V E F S V L G Q L P P G A T P L T S Q Y M N P Y G Y N I L W Q S K N G E A A N Q G M

2731 TAACATTTTC TCAAAATGAT TCAGCAATAA AAACATTTTA TTTGTACAAT ACTGAGGTGG TTCACCAAAC CACTGGCTCT CCTAGCCGCT GGAATGGTAA ATTTGATACT CTTTATGTCC AATCACCGAC

>...................................................................Cry65....................................................................>

V T F S Q N D S A I K T F Y L Y N T E V V H Q T T G S P S R W N G K F D T L Y V Q S P

2861 TTCCATTAAT CTTACACAAG GGTTTATTGT TATAGATACA ACAAAAAACA ACCCTGAACC ACCTATTGCA CTCCCCAACC AAGATATTTT AAGACCGTTT ACAAATGAAC ATGAAATATG GAATGGACGC

>...................................................................Cry65....................................................................>

T S I N L T Q G F I V I D T T K N N P E P P I A L P N Q D I L R P F T N E H E I W N G R

2991 TACTCTACAA AAGCACTTAA TCTATCTTTA TCAACTAGCT CAGGAGCCGA AGGGAAAATA AAGTTCTATA ATAATACTAA TTTAGTGCAT GAATCACCAG CGCTCTCCGG TTCTCCATCT GAACCTTATA

>...................................................................Cry65....................................................................>

Y S T K A L N L S L S T S S G A E G K I K F Y N N T N L V H E S P A L S G S P S E P Y

3121 CTTGGGAAGG CTCCTTTAAC CGAATAACAG TTTATCAAAG TAATAGTAGC GATAGCAATT ACTTTAATCT ATTCGGGGGA TTCCTTAAAC TCGATCCTAA TTCAAATGAC AACGGTAACA ATGGAGGTCC

>...................................................................Cry65....................................................................>

T W E G S F N R I T V Y Q S N S S D S N Y F N L F G G F L K L D P N S N D N G N N G G

3251 TCACAATGAT TGTGAGAATA TTGCTCACCC TGATCAACCA CTATATTACG AATCTAATAA TTTAATTTGG ACCGCCGCAC CTAACAAATT AGCATACTCT ACTACTGAAA TGCAGTTCGT TTTATCTGGC

>...................................................................Cry65....................................................................>

P H N D C E N I A H P D Q P L Y Y E S N N L I W T A A P N K L A Y S T T E M Q F V L S G

3381 GGCCTTGGAT TTACTCCTGG AGACATAGGT GCTAAAATCA AACTCAACTT CTGGAAGAAT GATTCTATAC AATACGTTGC ATACGGGGCA GTAGCAAATC TAGATTTCAT ATCTGCACCT GCACAATCGA

>...................................................................Cry65....................................................................>

G L G F T P G D I G A K I K L N F W K N D S I Q Y V A Y G A V A N L D F I S A P A Q S

3511 TACCTGGTGG ATTTGATAAA ATTACAATGG ACAACGACGA GTACAGTTTC TCGACCTCAC TTCGGATGAC GCTCGGCAGT GTATGTTACT AACACAAAAA GGAGGCCATG TAATGGCAAT ATCACGTATA

>...............................................Cry65..............................................>>

I P G G F D K I T M D N D E Y S F S T S L R M T L G S V C Y -

3641 ATCAATCCCA TTTTCACCAA ACAGGAACAT TTAGATCAAA TTACTAACCT TGTCAACAAT CTTTTTTCAT CGGGTAGCTC TTCTTTATCA CAGAGCGTCT CAGATTATTG GATCGATCAA GTTTTACTGA

3771 AGGTGAATGC ATTATCCGAT ACTGTATTTC CAACACAAAA AGAACAACTC CGTCAACGTC TTGCACAAGC AAAGCAAATC AGTAAAGCTC GTAATCTATT GGTAGGCGGG AATTTTGAAA CACTAAATAA

>>..................................................................ORF-1...................................................................>

M A L S D T V F P T Q K E Q L R Q R L A Q A K Q I S K A R N L L V G G N F E T L N

3901 GTGGAAATTG AGTCGGAATG CTGTTCTTGT AGCCGGCCAT GATCTTTTCC AAGGCTACCA TCTAGAATTA CCACCTGCCA TTGATTCTGT AAAATATCCT TCTTATGCCT ATCAAAAAAT AGATGAAAGT

>....................................................................ORF-1....................................................................>

K W K L S R N A V L V A G H D L F Q G Y H L E L P P A I D S V K Y P S Y A Y Q K I D E S

4031 AAATTAAAAG CCAATACACG TTACTATGTA TCAGCCTTTA TTGCACAAGG GGATCAACTT GAAATTATAG TATCCCGTTA TGGACAGGAG TACTCACAAA TCTTATATGT ACCAGCTGAA ATGGCAAAAC

>....................................................................ORF-1....................................................................>

K L K A N T R Y Y V S A F I A Q G D Q L E I I V S R Y G Q E Y S Q I L Y V P A E M A K

4161 CGATTTCTCC AGATGGAGGA CCAAATTGTT GTTCCCCTCA TCCGTGCAAT TGCGCAGCAT GTAATGAGGA AGAAGTAGAT TCGCATTTCT TCCAAGTTCC TATTGATGTA GGAACTTTAC AATCTTCTCA

>....................................................................ORF-1....................................................................>

P I S P D G G P N C C S P H P C N C A A C N E E E V D S H F F Q V P I D V G T L Q S S

4291 AAATCTAGGT ATTGAAATTG GTTTTAAAGT AGCAAGTACA GACGGATTTG CAAAACTTAG TAATATAGAA GTTTTCGAGG GCCGTCCATT AACTGCAGCA GAACAGCGAA AAGTATCACG CCTAGAAAAT

>....................................................................ORF-1....................................................................>

Q N L G I E I G F K V A S T D G F A K L S N I E V F E G R P L T A A E Q R K V S R L E N

4421 GAGTGGAAAG AGGAGCAACA AACCAAGGCG ACAGAACGCA CTCAGCTCCT CCAACAAATT CAGCAACGCT TCAATATGCT ATATACAACA CCAGAACACC ATACGTTACG CACAGAAACG AGCTACCAGC

>....................................................................ORF-1....................................................................>

E W K E E Q Q T K A T E R T Q L L Q Q I Q Q R F N M L Y T T P E H H T L R T E T S Y Q

4551 ATCTGCTAGA AACCATGCTT CCTTCTTTAC ACCATGTATA CCATTGGTTT ATGCCGGATG TACCAGATTC TGATTATGCC CTTTACTATG AGTTACAACA AAAGTTAGAA CGCGGATGGG ATCAATACTT

>....................................................................ORF-1....................................................................>

H L L E T M L P S L H H V Y H W F M P D V P D S D Y A L Y Y E L Q Q K L E R G W D Q Y

4681 CTCTCGAAAT CTCCTAGAGA ATGGAGATTT CTTAGAACCA CTTGATGATT CTTGGCATAC ACAAGGAACT GTTTCCCTTC ACACCATCAA CAACAATACA ATGTTACGAT TACAACATTG GGATTCCTTG

>....................................................................ORF-1....................................................................>

F S R N L L E N G D F L E P L D D S W H T Q G T V S L H T I N N N T M L R L Q H W D S L

4811 ATCCGCACAA ACGTATCCTT ACCAGTTGTC AATGAGAATG CTGAATATGT AATTCGAGTG ATTGGAAAAG GAACAGGAAG TGTACTCATC AAAAATGGAA CTATAACTAA CACACTTGCG TTTACAAACT

>....................................................................ORF-1....................................................................>

I R T N V S L P V V N E N A E Y V I R V I G K G T G S V L I K N G T I T N T L A F T N

4941 CAAGACAAAT GGAAACGAAG GAATTCCATT TACAACCAGA AAGGGAGCAA CTTTCGCTTA CTATACGTTC CGATGCAAAT GAATTTCTCG TAGATGCAAT CGAAGTCATC CTAATGAATG ATGGTGCCGA

>....................................................................ORF-1....................................................................>

S R Q M E T K E F H L Q P E R E Q L S L T I R S D A N E F L V D A I E V I L M N D G A

5071 AGAAGAAGAC CAGTTGCCAG GTATGTTTCC ACCTATAAAT TCTAACATGG GGTCTACACC GAACTCAAAC ATGATGAATA ACAATCAA**TA A**GTTTTAGGA AAGTGGGTGC AGGCGTTTGT CTGCACCTGC

**Terminator**

>...............................................ORF-1..............................................>>

E E E D Q L P G M F P P I N S N M G S T P N S N M M N N N Q -

5201 TTTTTTACAA AAAATCATTT TCAAGTTGAG ATATTTTATA TAGAAATATA TGGTATAAGA ATACACGGGG AGGTTTAATC ATGGTAAAGA AATCAAAAGT AATACAACAA AATACTCATG CAGCTTCAAC

5331 TACTACGTCT ACTACTGTAA CCCATCAAAA TCCTTCTTCC ACACCACCAG CAGGATATAA GAAGAAATCC GGCTGCGGTT GTGGAAAACG TCGTTGATTC CATGACATAG CTTTAGAAAT CACCTTATTC

5461 TTGGTAAGTT ACATGACTGG CTAATCATTC CTCTTAGCCG CTCTAAACTT ATAAACCCAA AGCCCATTGT TTCATCATTG CAATGGGCCT TTCTATCTTC ATTTTCTACA GGCTTCATTC TAACTGTACT

5591 ACTTTACGTA TACATAGGCT GCATTTGCTG TTACATAATA CGTCTTCCCC TTACTATTAT GCACCCTATA TTGTGAAAAA CCATTCAAAC TTACTTTTTT ATCCATTATG AATCC

**Fig. S1. Nucleotide sequence of the parasporin gene, *cry65Aa1*, and the deduced amino acid sequence.** The putative ribosome-binding site (RBS, boxed), BTⅠ (continuously underlined) and BTⅡ promoters (discontinuously underlined) are indicated as box in black font. The start and stop codons are boxed as red font. The arrows present the inverted sequence (IR) that is considered to be the terminator of *cry65Aa1*. Numbers on the sides are the numbers of nucleotide (upper) and amino acids (lower).


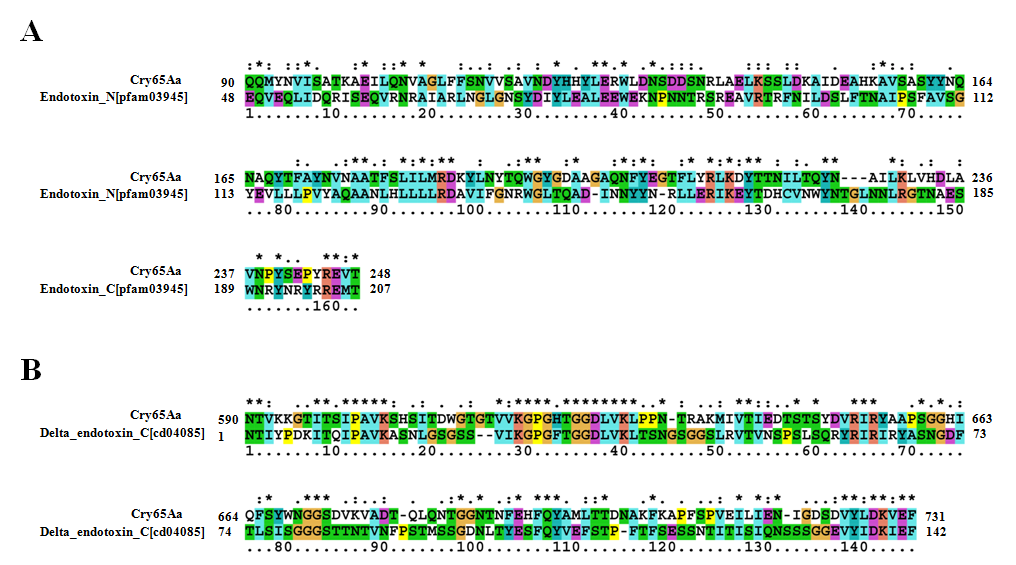


**Fig. S2. Cry65Aa1 is a novel holotype 3D-structure crystal protein.** There are two conserved domains which have high similarity with Endotoxin_N [pfam03945] (A) and Delta_endotoxin_C [cd04085] (B) in Cry65Aa1. The numbers on the bottom indicate the position of amino acids.


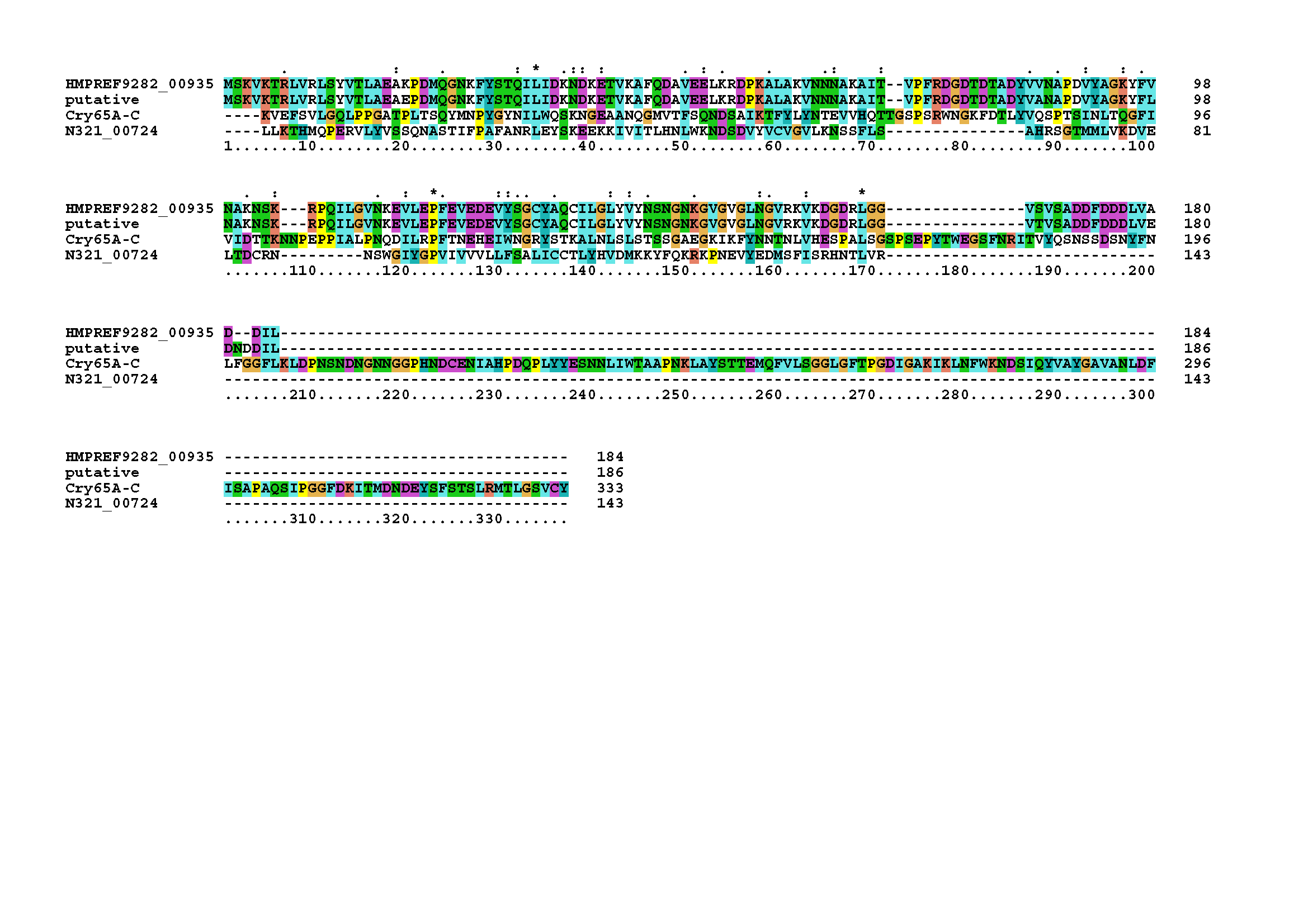


**Fig. S3. The Alignment of the shorter C-terminal regions of Cry65Aa1 with some high similarity selected proteins.** Cry65A-C means the shorter C-terminal regions of Cry65Aa1. The protein HMPREF9282_00935 means a hypothetical protein HMPREF9282_00935 from *Veillonella ratti* ACS-216-V-Col6b. The protein N321_00724 means a hypothetical protein N321_00724 from *Caprimulgus carolinensis*. The protein putative means a uncharacterized protein (WP_021841362.1) from *Veillonella* sp. CAG:933. The highly conserved residues are marked as * in position.

**
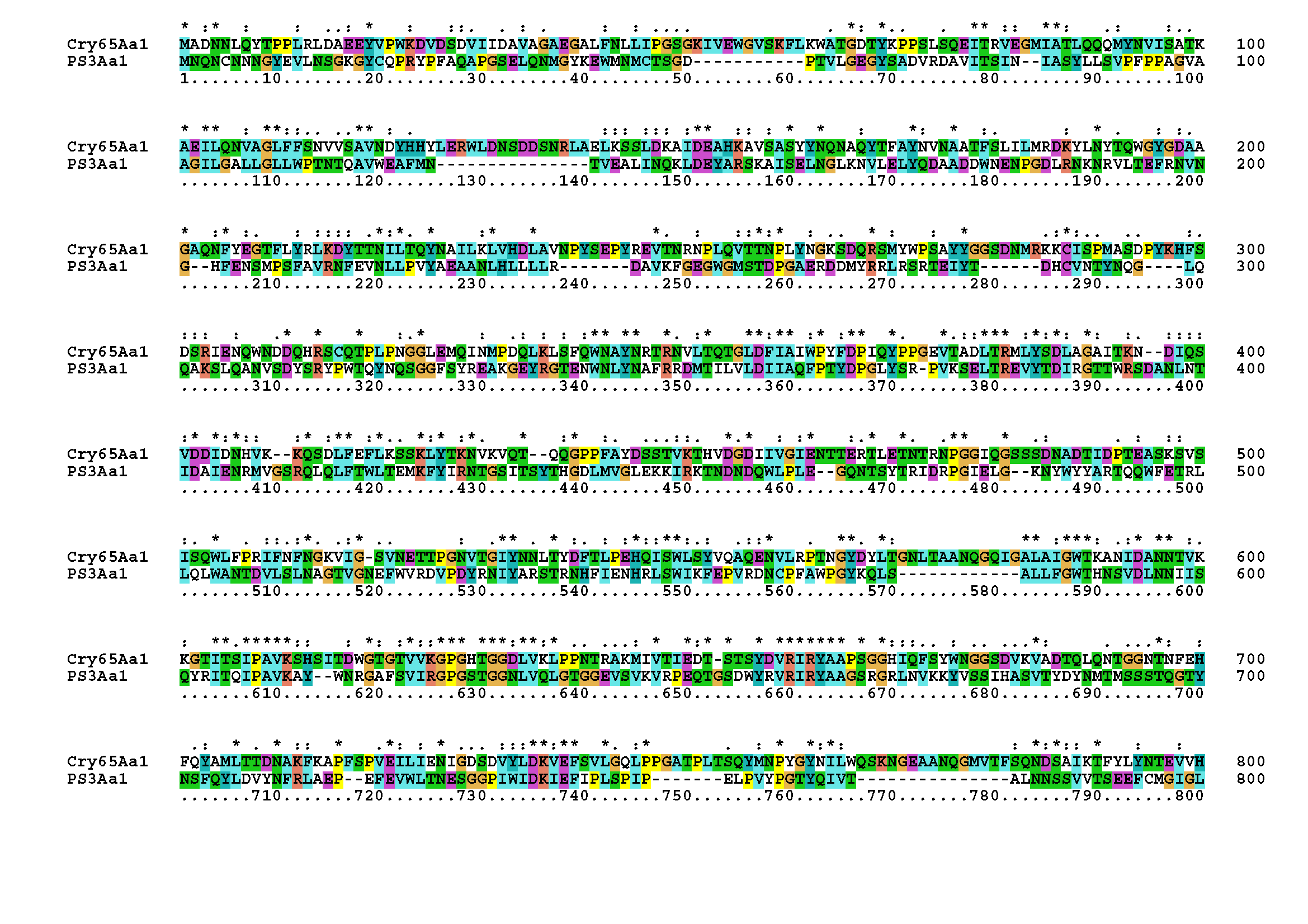
**

**
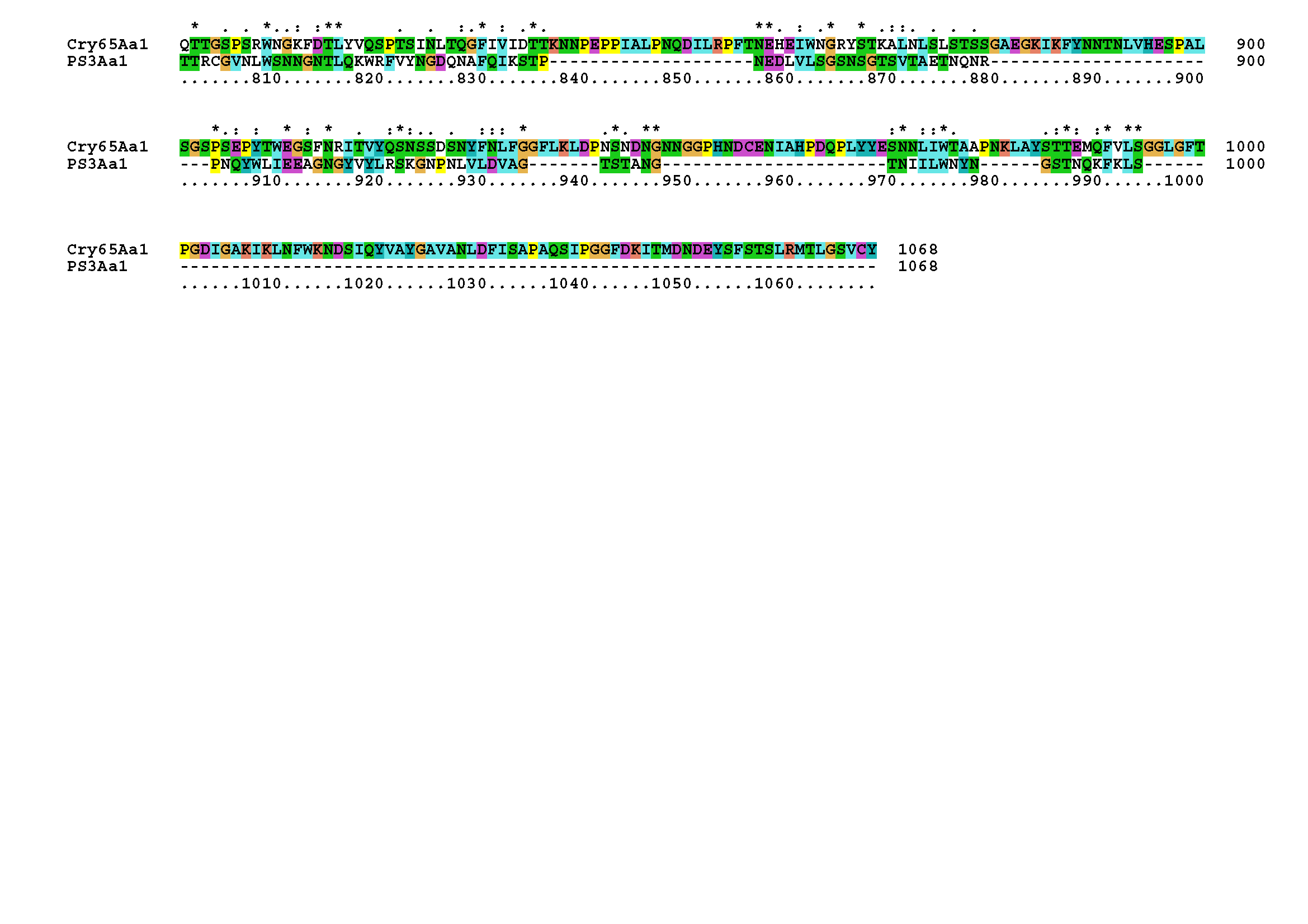
**

**Fig. S4. The Alignment of Cry65Aa1 amino acid sequence with that of Cry41Aa1 (PS3Aa1).** The highly conserved residues are marked as * in position.

**
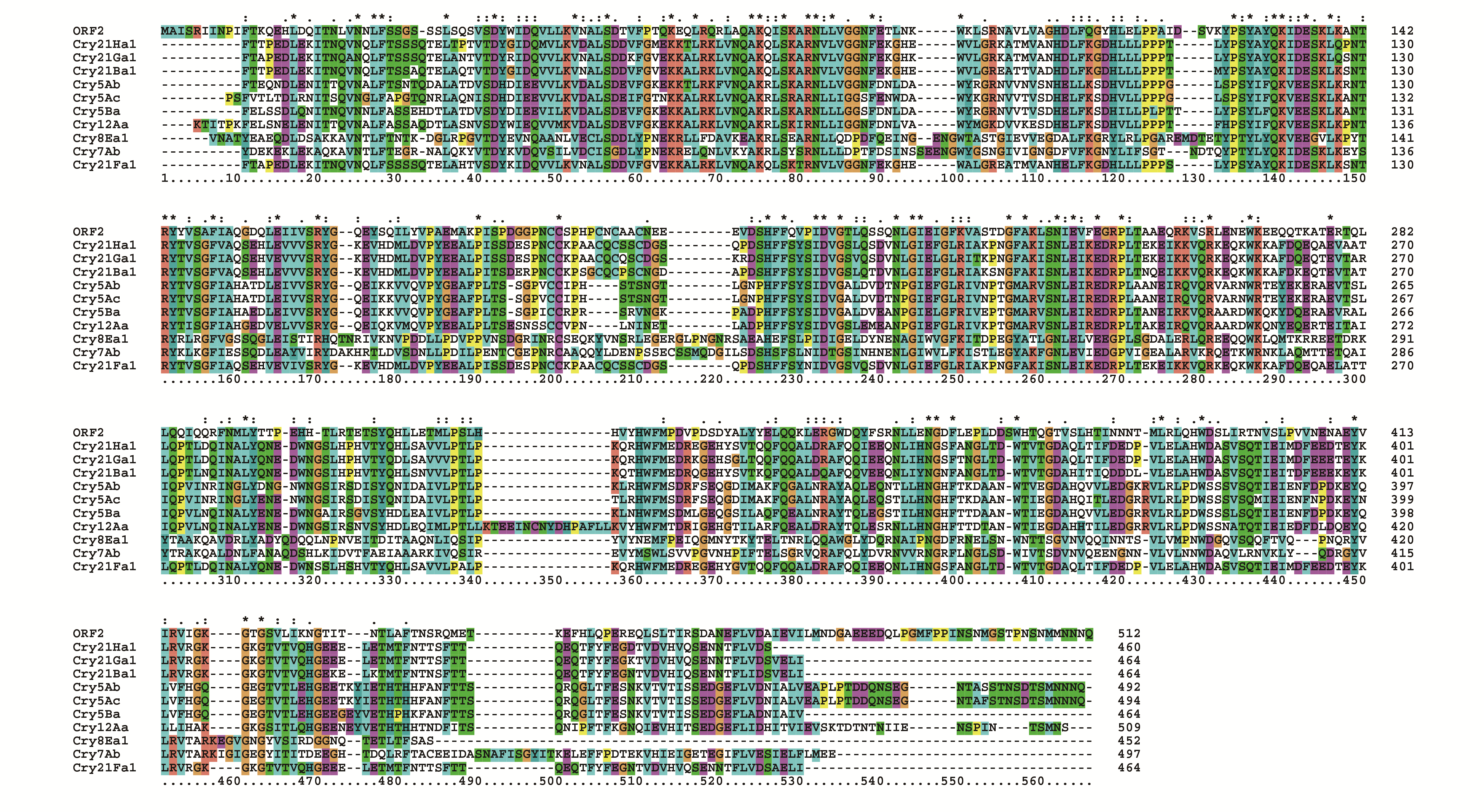
**

**Fig. S5. The Alignment of ORF2 amino acid sequence with the C-terminal regions of some high similarity selected Cry proteins.** The highly conserved residues are marked as * in position.

**
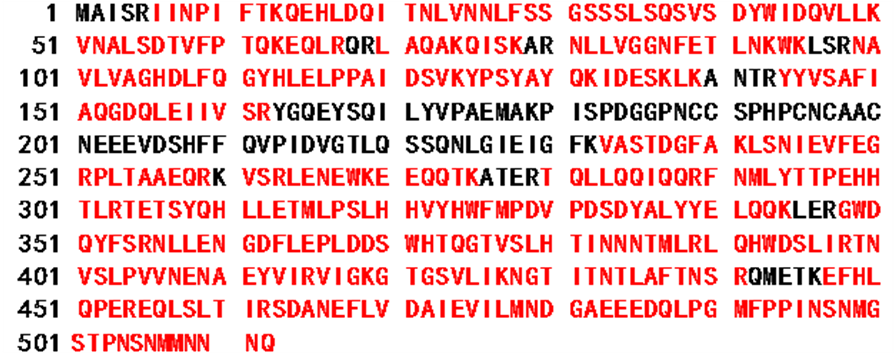
**

**Fig. S6. The MALDITOF/TOF-MS results of 58 kDa protein band in Cry65Aa1 crystals.** The matched peptides shown in Bold Red.

**Tables**

**Table S1. Bacterial strains and plasmids used** in this study.

| Strains or plasmids | Characteristics | Origin or references |
| --- | --- | --- |
| ***Escherichia coli* strains** | | |
| DH5α | *supE44 lacU_169_* (*φ80 lacZ M15*) *Hsd R17 recA1 end A1 gyr A_96_ thi^-1^ relA1* | Stored in this lab |
| EMB1330 | DH5α derivative with plasmid pEMB1330 | This work |
| ***Bacillus thuringiensis* strains** | | |
| BMB171 | Acrystalliferous mutant, subsp. | Stored in this lab |
| SBT-003 | wild type strains isolated in China soil sample | Stored in this lab |
| **Plasmids** | | |
| pUC18-T | A clone vector, ori*E. coil,* Amp^r^, 2.7 kb | Takara |
| pHT304 | A *E. coil* to *Bt* shuttle vector, Amp^r^, Erm^r^, 6.6 kb | [^1^](#_ENREF_1) |
| pBMB1A | A *E. coil* to *Bt* shuttle vector, harbored BtI-BtII promoter, SD sequence and terminator of Cry1Ac, Amp^r^, Erm^r^, 7.9 kb | [^2^](#_ENREF_2) |
| pEMB1330 | Derivative of pUC18-T, containing a 3.2 kb *cry65Aa1* gene, Amp^r^, 5.9 kb | This work |
| pBMB1A-65A | Derivative of pBMB1A, containing a 3.2 kb *cry65Aa1* ORF, Amp^r^, Erm^r^, 11.1 kb | This study |
| pBMB1331 | Derivative of pHT304, containing a 5.3 kb genome fragment of SBT-003, which harbored *cry65Aa1* and *orf2* gene under the control of promoter and terminator of Cry65Aa, Amp^r^, Erm^r^, 11.9 kb | This work |
| pBMB1A-65Opn | Derivative of pHT304, containing a 4.7 kb genome fragment of SBT-003, which harbored *cry65Aa1* and *ofr2* gene, Amp^r^, Erm^r^, 12.6 kb | This work |
| pBMB1332 | Derivative of pHT304, containing a 3.5 kb fragment of *cry65Aa1* and its promoter, and 0.2 kb terminator of Cry65Aa , Amp^r^, Erm^r^, 10.3 kb | This work |
| pBMB1333 | Derivative of pHT304, containing a 1.5 kb fragment of *orf2*, 0.4 kb promoter, and 0.2 kb terminator of Cry65Aa, Amp^r^, Erm^r^, 8.7 kb | This work |
| pBMB1A-ORF2 | Derivative of pBMB1A, containing a 1.5 kb fragment of *orf2*, Amp^r^, Erm^r^, 9.4 kb | This work |
| pBMB1334 | Derivative of pBMB1331, has a mutation from ATG to TAA at the original start code site of ORF2, Amp^r^, Erm^r^, 11.9 kb | This work |
| pBMB1335 | Derivative of pBMB1331, has a mutation which removed the stem-and-loop structure form 4254-4277 site of Cry65Aa operon, Amp^r^, Erm^r^, 11.9 kb | This work |
| pBMB1336 | Derivative of pBMB1331, has truncated *orf2*, retains the fragment from ATG to 4300 site of Cry65Aa operon, Amp^r^, Erm^r^, 10.9 kb | This work |
| pBMB1337 | Derivative of pBMB1331, has truncated *orf2*, retains the fragment from ATG to 4300 site and the terminator of Cry65A operon, Amp^r^, Erm^r^, 11.1 kb | This work |
| pBMB21B-N::ORF2 | Derivative of pHT304, containing the Cry21Ba N terminus (Cry21Ba-N) and *orf2* in frame under the control of promoter and terminator of Cry65Aa | This work |
| pBMB65A-N::1Ac-C | Derivative of pHT304, containing the Cry65A N terminus (Cry65A-N) and the C-terminal domain of Cry1Ac in frame under the control of promoter and terminator of Cry65Aa | This work |
| pBMB65A-N::ORF2 | Derivative of pHT304, containing the Cry65A N terminus (Cry65A-N) and ORF2 in frame under the control of promoter and terminator of Cry65Aa | This work |
| pBMB65A-N::21Ba-C | Derivative of pHT304, containing the Cry65A N terminus (Cry65A-N) and Cry21Ba in frame under the control of promoter and terminator of Cry65Aa | This work |
| pBMB1338 | Derivative of pBMB65A-N::ORF2, containing the Cry65A N terminus (Cry65A-N) and the C-terminal domain of Cry1Ac in frame, and ORF2 in an operon under the control of promoter and terminator of Cry65Aa | This work |
| pBMB1339 | Derivative of pBMB1332, containing the Cry65A and Cry1Ac-C in an operon under the control of promoter and terminator of Cry65Aa | This work |
| pBMB1340 | Derivative of pBMB1332, containing the Cry65A and Cry21Ba-C in an operon under the control of promoter and terminator of Cry65Aa | This work |

**Table S2.** **Primers used in this study.**

| **Primers** | **Sequences (5’-3’)^a^** |
| --- | --- |
| 65A-F | CGCGGATCCTTGGCAGATAATAATTTACA |
| 65A-R | CCGCTCGAGTTAGTAACATACACTGCCGA |
| 65Opn-F | GCCGGATCCCATCTGCAAAGACAATAGAC |
| 65Opn-R | GGCAAGCTTGAAGGATTTTGATGGGTTAC |
| ORF2-F-1 | CGCGGATCCATGGCAATATCACGTATAAT |
| ORF2-R-1 | CCGCTCGAGTTATTGATTGTTATTCATCA |
| P65-R | CTTTTAAGGAGGAATTATTATGCATTATCCGATACTG |
| ORF2-F-2 | CAGTATCGGATAATGCATAATAATTCCTCCTTAAAAG |
| T65-F | CCGCTCGAGTTAGTAACATACACTGCCGAG |
| Morf2-1 | GAAATACAGTATCGGATAATGTTATCACCTTCAGTAAAACTTG |
| Morf2-2 | CAAGTTTTACTGAAGGTGATAACATTATCCGATACTGTATTTC |
| Morf2-3 | CCGCTCGAGGGAAGAAATGCGAATCTACTT |
| Morf2-4 | CCGCTCGAGTACAATCTTCTCAAAATCTAG |
| Morf2-5 | CCGCTCGAGACCTAGATTTTGAGAAGATTG |
| 65AN-R | CCGGTCGACAACTCCACTTTATCCAGGTAC |
| 1Ac-C-F | TTAGTCGACATTCCAGTTACTGCAACA |
| 1Ac-C-R | ACAGGATCCCCCTATTAGTGCTTTATTT |
| ORF2-F-3 | CCGGTCGACATGGCAATATCACGTATAAT |
| 21Ba-C-F | ACGCGTCGACTCTACTTCTACTCCTACTTC |
| 21Ba-C-R | CCCGGATCCCTTGGTTTAAAAAACCAATA |
| 21Ba-N-F | CGCGGATCCTCAATCCTTGAGGGGCTTCC |
| 21Ba-N-R | ACGCGTCGACAGGGAGAGAAATAAATTCAA |
| Coe-F | GGCATGGACCACCTAAACTATTTTAATC |
| Coe-R | CCAATAAATCACAAAGTTTTAAGGGCGG |
| Qrt-F | GGCAGATAATAATTTACAGTATACACC |
| Qrt-R | TACCTGAGCCTGGAATTAATAGATTG |

^a^ The underline showed the restriction enzyme sites.

1 Arantes, O. & Lereclus, D. Construction of cloning vectors for Bacillus thuringiensis. *Gene* **108**, 115-119 (1991).

2 Zheng, W., Ye, W., Peng, D. & Sun, M. Construction of Bacillus thuringiensis Expression Vector by Using Regulatory Elements from cry1Ac gene. *Hubei Agricultural Sciences* **51**, 6 (2012).
